# CRISPR/Cas9‐based editing of *NF‐YC4* promoters yields high‐protein rice and soybean

**DOI:** 10.1111/nph.20141

**Published:** 2024-09-22

**Authors:** Lei Wang, Seth O'Conner, Rezwan Tanvir, Wenguang Zheng, Samuel Cothron, Katherine Towery, Honghao Bi, Evan E. Ellison, Bing Yang, Daniel F. Voytas, Ling Li

**Affiliations:** ^1^ Department of Biological Sciences Mississippi State University Mississippi State MS 39762 USA; ^2^ College of Life Sciences Shihezi University Shihezi Xinjiang 832003 China; ^3^ Department of Genetics, Development and Cell Biology Iowa State University Ames IA 50011 USA; ^4^ Department of Genetics, Cell Biology and Development, Center for Genome Engineering University of Minnesota Minneapolis MN 55108 USA; ^5^ Division of Plant Science and Technology, Bond Life Sciences Center University of Missouri Columbia MO 65211 USA; ^6^ Donald Danforth Plant Science Center St Louis MO 63132 USA

**Keywords:** CRISPR/Cas9, NF‐YC4 promoter, protein, rice, soybean

## Abstract

- Genome editing is a revolution in biotechnology for crop improvement with the final product lacking transgenes. However, most derived traits have been generated through edits that create gene knockouts.
- Our study pioneers a novel approach, utilizing gene editing to enhance gene expression by eliminating transcriptional repressor binding motifs.
- Building upon our prior research demonstrating the protein‐boosting effects of the transcription factor NF‐YC4, we identified conserved motifs targeted by RAV and WRKY repressors in the *NF‐YC4* promoters from rice (*Oryza sativa*) and soybean (*Glycine max*). Leveraging CRISPR/Cas9 technology, we deleted these motifs, resulting in reduced repressor binding and increased *NF‐YC4* expression. This strategy led to increased protein content and reduced carbohydrate levels in the edited rice and soybean plants, with rice exhibiting up to a 68% increase in leaf protein and a 17% increase in seed protein, and soybean showing up to a 25% increase in leaf protein and an 11% increase in seed protein.
- Our findings provide a blueprint for enhancing gene expression through precise genomic deletions in noncoding sequences, promising improved agricultural productivity and nutritional quality.

## Abstract 2

See also the Commentary on this article by Wang & Niyogi, **245**: 1805–1807.

## Introduction

Mutations that cause a loss‐of‐function or a gain‐of‐function are pivotal tools for elucidating the roles of genes as well as modulating biological traits. The advent of CRISPR‐based editing has revolutionized biotechnology, offering high efficiency and high specificity for developing gene‐edited, transgene‐free crops (Qi *et al*., 2013; Gilbert *et al*., 2014; Shan *et al*., 2014; Chavez *et al*., 2015, 2016; Konermann *et al*., 2015; Li *et al.*, 2012, 2017; Hu *et al*., 2018; Kwon *et al*., 2020; Lin *et al*., 2020; Lu *et al*., 2020). In agriculture, three primary genome editing types are prominent: Site‐directed nuclease‐1 (SDN‐1), resulting from nonhomologous end joining (NHEJ) repair at nuclease cut sites (EFSA, 2012; Podevin *et al*., 2013; Hsu *et al*., 2014; Bortesi & Fischer, 2015); SDN‐2, via homology‐dependent repair (HDR) using a specific repair template (Sonoda *et al*., 2006); and SDN‐3, involving exogenous DNA insertion at cut sites via HDR or NHEJ (EFSA, 2012). SDN‐1 products generally encounter higher public acceptance and fewer regulatory barriers globally.

Our objective is to utilize an SDN‐1 approach with the CRISPR/Cas9 system to edit promoter repressor elements, thereby boosting the expression of target genes and developing transgene‐free crops with desirable agronomic traits. Within our efforts to meet the rising demand for protein amidst a growing global population (Lucas *et al*., 2015), our previous research demonstrated that overexpressing the Arabidopsis orphan gene Qua‐Quine Starch (*QQS*) enhanced leaf and seed protein levels in multiple plant species by interacting with Nuclear Factor Y subunit C4 (NF‐YC4) genes (Li *et al*., 2009, 2015; Li & Wurtele, 2015). The conserved functionality of *NF‐YC4* across diverse plant species was evident, as demonstrated by similar phenotypes upon overexpressing orthologs in soybean and maize (O'Conner *et al*., 2018).

Here, we illustrated that transgenic overexpression of *OsNF‐YC4* in rice and *GmNF‐YC4* in soybean enhances leaf and seed protein content while reducing starch accumulation. Furthermore, we developed a novel approach using a CRISPR/Cas9‐based method to target a noncoding regulatory motif, and successfully used it to engineer *NF‐YC4* promoters with deletions of transcriptional repressor binding motifs. These targeted genomic edits in rice and soybean effectively diminished repressor binding, elevated *NF‐YC4* expression, and consequently boosted protein levels and reduced starch content. This innovative approach offers promising applications for enhancing gene expression through precise genomic alterations, paving the way for improved crop productivity and nutritional value.

## Materials and Methods

### Plant materials and growth conditions

Seeds of the rice (*Oryza sativa* L. ssp. *japonica*) cultivar Kitaake were germinated on moist paper towels in Petri dishes at 30°C in an oven. Germinating seeds were transferred to pots containing soil (Pro‐Line C/25 soil mix) and placed in a growth chamber (Model LT105; Percival Scientific, Perry, IA, USA). The growth chamber conditions were set to 28°C in the light and 25°C in the dark, with a photoperiod of 16 h : 8 h, light : dark. *OsNF‐YC4‐OE* plants were grown in growth chamber. After 2 wk in the growth chamber, selected *OsNF‐YC4‐promoter‐CRISPR* plants were transplanted to Mississippi State University North Farm for field cultivation.

For soybean (*Glycine max* L.) cultivar Williams 82, *GmNF‐YC4‐1‐promoter‐CRISPR* and null‐segregant plants were cultivated at the Mississippi State University North Farm.

Spraying of the herbicide solution (0.0735% glufosinate ammonium and 0.1% Tween 20) and PCR of the *BAR* gene were employed to identify *OsNF‐YC4‐OE* (Os3g14669) rice plants and *GmNF‐YC4‐1‐promoter‐CRISPR* (Glyma06g17780) soybean plants. Additionally, PCR assays for T‐DNA were used to identify *OsNF‐YC4‐promoter‐CRISPR* and *GmNF‐YC4‐1‐promoter‐CRISPR* rice and soybean plants. Sequences of the oligos used as primers are provided in Supporting Information Table S1.

### Constructs and transformation

For the overexpression of *OsNF‐YC4* in rice, the *35S:OsNF‐YC4* construct in the pB2GW7 vector was utilized, as previously described (Li *et al*., 2009, 2015). This construct was introduced into rice using the *Agrobacterium tumefaciens* strain EHA101.

To create the construct for Cas9 and the single guide RNAs (sgRNAs) for *OsNF‐YC4‐promoter‐CRISPR*, a protocol similar to previously described methods was employed (Zhou *et al*., 2014). Initially, two reverse‐complementary DNA oligos with unique 4‐base overhangs were designed to produce a single sgRNA matching the targets of interest. Subsequently, the two oligos were annealed, and the resulting double‐stranded fragments (dsOligo) were ligated into the *Btg*ZI‐predigested pgRNA1, resulting in the first gRNA. The second gRNA was sequentially constructed by inserting another dsOligo at the *Bsa*I sites. The gRNA cassette, expressing two guides, was then mobilized into the destination vector pCas9‐GW through Gateway™ cloning reactions. The binary plasmid, expressing Cas9 under the *Zm*Ubi promoter, gRNAs under U6 promoters, and the hygromycin resistance gene under the 35S promoter, was introduced into the *Agrobacterium* strain EHA105 for rice transformation.

The soybean CRISPR construct (*GmNF‐YC4‐1‐promoter‐CRISPR*) was generated using a method as previously described (Čermák *et al*., 2017; Curtin *et al*., 2018). In brief, three binary vectors, namely pSC218GG, pSC218UG, and pSC218RG, were utilized for the delivery of the CRISPR/Cas9, as previously described (Curtin *et al*., 2018). The construct for Cas9 was assembled, and guide RNAs for each target were engineered using a PNK oligo annealing assay and cloned into three binary vectors using Gateway™ cloning reactions, following established procedures. For the transformation, the disarmed *A. rhizogenes* strain 18r12 was employed for whole‐plant transformation as previously reported (Curtin *et al*., 2018). The oligo information is provided in Table S1.

The resulting constructs were used for rice and soybean transformation at the Iowa State University Plant Transformation Facility (PTF). For rice transformation, the cultivar Kitaake was employed for both *OsNF‐YC4‐OE* and *OsNF‐YC4‐promoter‐CRISPR* experiments. The soybean cultivar Williams 82 was used for *GmNF‐YC4‐1‐promoter‐CRISPR* studies. Following transformation, the transformed materials were obtained from the PTF as either the T0 generation plants (for rice) or T1 generation seeds (for soybean). These plants and seeds served as the starting material for further analyses and experiments.

### PLACE program analysis

The *cis*‐acting regulatory DNA elements in the promoter regions of *OsNF‐YC4* and *GmNF‐YC4‐1* were analyzed using two primary tools: the PLACE program (Higo *et al*., 1999) and JASPAR (Rauluseviciute *et al*., 2024) as previously described. PLACE (Plant *cis*‐acting regulatory DNA elements) was used to identify plant‐specific *cis*‐regulatory motifs within the promoter sequences of *OsNF‐YC4* and *GmNF‐YC4‐1*. The sequences were input into the PLACE database search tool, using default parameters to identify conserved motifs. Specific transcription factor binding motifs, particularly those linked to RAV1A and WRKY transcription factors, were identified by aligning the promoter sequences with JASPAR's curated plant transcription factor binding profiles. PLACE provided insights into plant‐specific motifs, while JASPAR offered detailed models of transcription factor binding. Together, these analyses comprehensively identified RAV1A and WRKY binding motifs crucial for the regulation of *OsNF‐YC4* and *GmNF‐YC4‐1*.

### Luciferase reporter assay

The luciferase reporter assay was conducted according to procedures previously described (Hellens *et al*., 2005). Briefly, PCR was used to introduce deletions into the promoters of *OsNF‐YC4* and *GmNF‐YC4‐1*, respectively. The full‐length promoter and its deletion derivatives were aligned using ClustalW (http://www.ebi.ac.uk/Tools/clustalw2/index.html) to visualize the deletions. Subsequently, the promoter fragments were cloned into a pUC19‐derived vector, where they were fused to the luciferase reporter gene. These constructs were then transformed into *Agrobacterium* and transiently expressed in tobacco (*Nicotiana benthamiana* L.) leaves following infiltration.

Controls were included, which consisted of the full‐length *OsNF‐YC4* or *GmNF‐YC4‐1* promoter recombined with the luciferase reporter gene. d‐fluorescein (1 mM) was sprayed onto the tobacco leaves, and images were captured using a NightOWL II LB983 fluorescence system. The relative luminescence intensity was calculated using Image‐Pro Plus 6.0 (Media Cybernetics, Silver Spring, MD, USA). Each luciferase reporter assay experiment was repeated at least three times.

### DNA isolation and PCR sequencing

DNA was isolated using the CTAB method (Li *et al*., 2015; Qi *et al*., 2019). For PCR, the DreamTaq kit (Thermo Fisher Scientific, Waltham, MA, USA) was employed to determine deletions in the promoter and to determine if vector sequence was present in the plants. The PCR product from all individual CRISPR plants were subjected to sequencing by Genewiz (South Plainfield, NJ, USA) to confirm the presence of desired edits and to verify the integrity of the edited sequences.

### RNA isolation and real‐time PCR

Young leaves or single seeds of rice and soybean plants were collected, and total RNA was extracted using TRIzol reagent (Invitrogen™) following the manufacturer's instructions (Qi *et al*., 2019). Complementary DNA (cDNA) was synthesized using M‐MuLV Reverse Transcriptase (New England Biolabs, Ipswich, MA, USA). Subsequently, quantitative real‐time polymerase chain reaction was performed using PowerUp™ SYBR™ Green Master Mix (Thermo Fisher Scientific) on an Applied Biosystems™ StepOnePlus™ Real‐Time PCR System (Thermo Fisher Scientific), following the manufacturer's instructions. The rice tubulin *beta‐7 chain‐like* gene (Os03g0780600) was used as the internal control for rice samples (Yoshikawa *et al*., 2003), and the soybean *actin* gene (Glyma.15g050200) was used as the internal control for soybean samples (O'Conner *et al*., 2018; Qi *et al*., 2019). Relative expression was determined using the 2^−ΔΔCt^ method. Each quantitative real‐time polymerase chain reaction assay was conducted with at least three technical replicates. Primer sequences are listed in Table S1.

### Composition analysis

Seedlings and seeds of rice and soybean were harvested for leaf starch and protein tests at the end of the light period from plants grown in soil in the growth chamber or in the field at 30 d after planting. I_2_/KI staining for starch was conducted as previously described (Li *et al*., 2007, 2009). Starch quantification was performed using the GOPOD reagent assay (Li *et al*., 2009). Determination of protein content was conducted using the Pierce™ Modified Lowry Protein Assay Kit (Thermo Fisher Scientific) (Li *et al*., 2015; O'Conner *et al*., 2018; Qi *et al*., 2019). These experiments were repeated three times with similar results. Each experiment included three biological replicates.

Soybean seed protein was analyzed via near‐infrared spectroscopy (NIRS) using an Infratech NOVA grain analyzer (FOSS North America, Eden Prairie, MN, USA), including three biological replicates.

### Electrophoretic mobility shift assay (EMSA)

Different DNA fragments were obtained by PCR from WT‐sibling plants and CRISPR/Cas9‐edited plants. Biotin labeling of the DNA fragments was performed using the Pierce™ Biotin 3′‐End DNA Labeling Kit (Thermo Fisher Scientific), followed by purification of both biotin‐labeled and unlabeled DNA fragments using the QIAquick PCR Purification Kit (Qiagen).

Protein expression constructs were generated using the Gateway system. Coding sequences of *OsWRKY121* (Os03g0741400), *OsWRKY71* (Os02g0181300), *OsRAV3* (Os01g0693400), *GmRAV1* (Glyma.01G087500), and *GmWRKY27* (Glyma.03 g224700) were amplified by PCR, inserted into the pDONR221 vector (Invitrogen™) using BP Clonase II (Thermo Fisher Scientific), and transferred to the destination vector pDEST‐HisMBP (Nallamsetty *et al*., 2005) using LR Clonase II (Thermo Fisher Scientific). Sequence‐confirmed plasmids were transformed into BL21 (DE3) cell line for protein expression. Protein expression was induced by 0.5 mM IPTG at 18°C overnight. The protein was purified using Ni‐NTA agarose (Invitrogen™). Protein content was determined using the Bradford assay with bovine serum albumin as the standard (Bio‐Rad).

Assays followed the protocol of the LightShift™ Chemiluminescent EMSA Kit (Thermo Fisher Scientific) with biotin‐labeled DNA. The EBNA extract and unlabeled control DNA were provided as positive controls.

### Statistical analyses

A minimum of three biological determinations from each independent transgenic line and each control were used for qualitative and quantitative analyses of composition. For compositional analyses, data are presented as mean ± SEM (Standard Error of Mean). Statistical significance relative to the control was calculated with Student's *t*‐test.

## Results

### *OsNF‐YC4* overexpression enhances leaf and seed protein content in rice

**Fig. 1:**
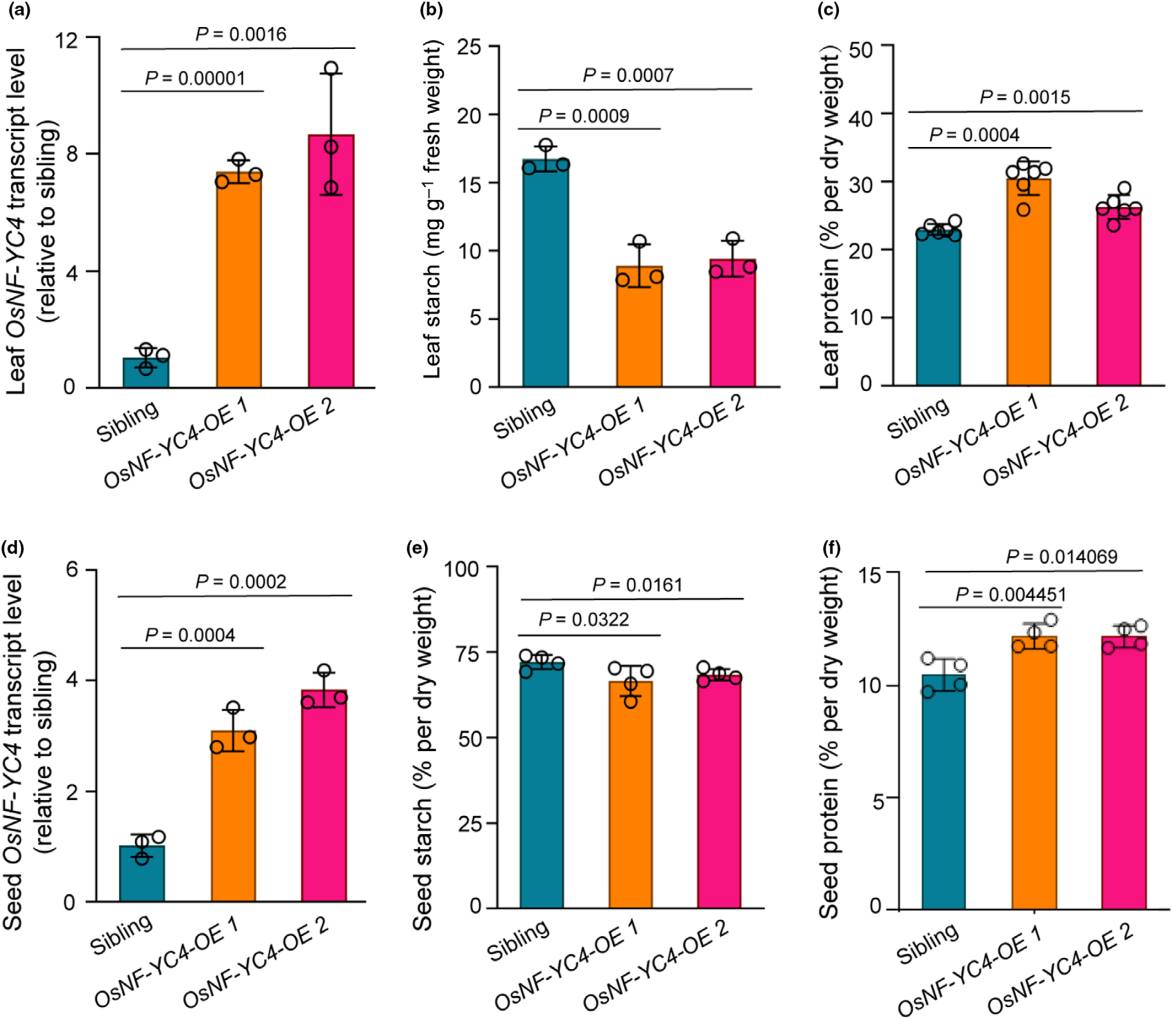
Overexpression of the *OsNF‐YC4* gene increased protein content in rice plants. (a, d) *OsNF‐YC4* transcript levels were significantly increased in *OsNF‐YC4‐OE* rice plants in leaf (a) and in seed (d), relative to sibling, the value of which was set to 1. (b, e) Leaf and seed starch levels were notably decreased in transgenic events with increased *OsNF‐YC4* expression. (c, f) Both leaf and seed protein contents were increased in *OsNF‐YC4* overexpression lines. Individual transgenic plants and siblings were identified by both herbicide resistance and PCR screening of leaf genomic DNA. Leaf samples were collected from the middle third of the second leaf from the primary tiller of T3 plants at the end of the light period 30 d after planting, while mature T4 seeds were analyzed, from growth chamber. Each analysis was conducted in triplicate, using 10 plants per line. All values represent mean values ± error bars indicating the SE of mean; *n*  ≥ 3. Student's *t*‐test was used to compare the *OsNF‐YC4‐OE* plants with sibling controls.

To validate the functional consistency of NF‐YC4 in modulating protein and starch content across different crops, we measured leaf and seed protein levels in transgenic *35S:OsNF‐YC4* (Os03g14669) rice plants. Rice, a staple crop with limited protein content, was chosen as the experimental model. Our analysis of the *OsNF‐YC4* overexpression lines revealed a substantial increase in *OsNF‐YC4* transcript levels, reaching 7–9 times higher in leaves (Fig. 1a) and 3–4 times higher in seeds (Fig. 1d), compared to null‐segregant siblings (wild‐type (WT) siblings) (*P* < 0.01).

In *35S:OsNF‐YC4* rice plants, we observed a significant reduction in leaf starch levels, by 43.7–46.8% (*P* < 0.01), and seed starch levels, by 3.5–4.8% (*P* < 0.05), compared to WT siblings (Fig. 1b,e). Concurrently, there was a substantial increase in leaf protein content, by 17.5%–29.9% (*P* < 0.05), and seed protein levels, by 18.4–21.4% (*P* < 0.1), relative to WT siblings (Fig. 1c,f). However, there was no significant difference in plant or seed growth, development or appearance (Fig. S1a), in plant height, panicle number per plant, seed number per panicle, and seed weight per seed or per plant compared to WT siblings (Fig. S1b) (*P* > 0.1). These findings highlight the ability of ectopic *OsNF‐YC4* overexpression to substantially enhance protein content in both leaves and grains of transgenic rice plants, without impacting the yield.

### Transcriptional suppression of the *NF‐YC4* promoter by RAV and WRKY transcription factors

Predictive analysis of *cis*‐acting regulatory elements within the *OsNF‐YC4* promoter and *GmNF‐YC4‐1* promoter using the PLACE (Higo *et al*., 1999) and JASPAR (Rauluseviciute *et al*., 2024) databases identified two classes of conserved binding sites. The sequences TGTTG/CAACA for RAV1A (Related to ABI3/VP1 1, ABSCISIC ACID INSENSITIVE 3/Viviparous1) and RGTCA/TGACY (W‐box motif) for WRKY transcription factors were detected not only in the *OsNF‐YC4* promoter but also in the promoter of *GmNF‐YC4‐1* in soybean (Figs S2, S3). RAV1As and WRKYs are known to function as repressors that dampen gene transcription (Zhang *et al*., 2004; Rushton *et al*., 2010; Wang *et al*., 2015; Shin & Nam, 2018; K. Zhang *et al.*, 2019).

**Fig. 2:**
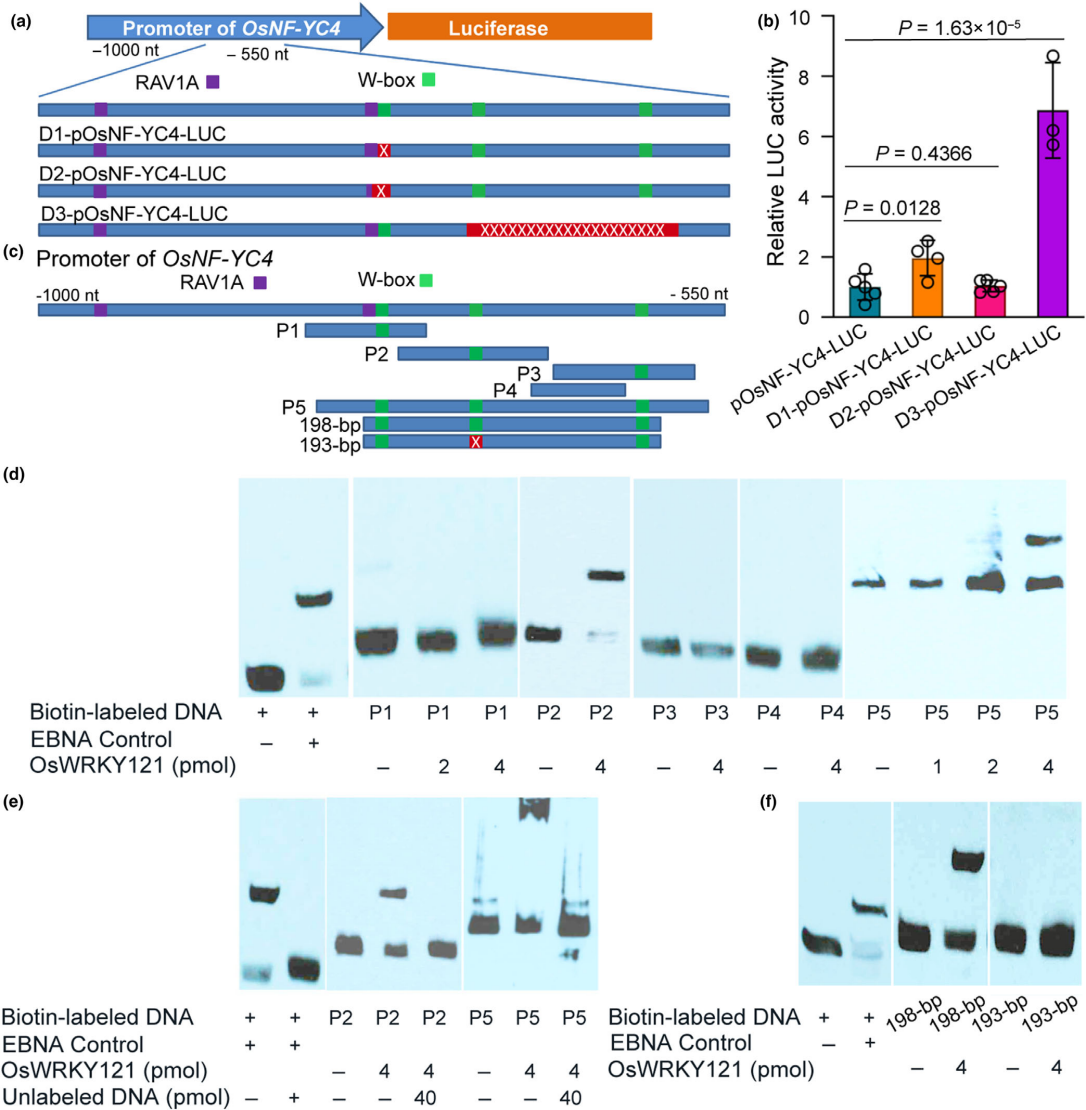
The second W‐box in the *OsNF‐YC4* promoter region is bound by OsWRKY121, and its deletion increased *OsNF‐YC4* transcription. (a) Structure of the full‐length *OsNF‐YC4* promoter and deletion derivatives used for luciferase assays. RAV1A (CAACA/TGTTG) and WRKY (RGTCA/TGACY) binding sites are indicated as purple and green boxes, respectively. Deleted regions are in red with a single or string of ‘x's. (b) Relative luciferase activity (normalized against total protein content) increased upon deletion of the second and third WRKY binding sites. Promoter versions D1, D2, and D3 were fused to luciferase reporter genes and transiently expressed in tobacco leaves after infiltration. Bar graphs show mean values ± error bars indicating the SE of mean; *n*  ≥ 3 independent experiments. Student's *t*‐test was used to compare the LUC (luciferase) activity between deletions and controls. (c) The structures of *OsNF‐YC4* promoter fragments (P1–P5, 198‐bp, and 193‐bp) with different combinations of W‐box motifs. These fragments were PCR‐amplified from WT plants and used for biotin labeling and EMSA (Electrophoretic Mobility Shift Assay) in (d–f). (d) EMSA demonstrated that OsWRKY121 could bind to the second W‐box (in P2) of the *OsNF‐YC4* promoter, but not to the first (in P1) or the third (in P3) W‐box motifs. P4 served as a negative control without any W‐box, while P5 contained all three W‐box motifs. (e) Competition with unlabeled fragments confirmed the specific binding of OsWRKY121 to the second W‐box motif (P2) in the *OsNF‐YC4* promoter. (f) OsWRKY121 bound to the 198‐bp native fragment containing all three W‐box motifs, but not the 193‐bp synthesized fragment lacking the second 5‐bp W‐box (shown in a red box in c), confirming the importance of the second W‐box for OsWRKY121 binding. Twenty femtomole of biotin‐labeled DNA fragments were used in (d–f). EBNA (Epstein–Barr nuclear antigen) from the EMSA kit served as a positive control in (d–f).

Within the *OsNF‐YC4* promoter, we identified two RAV1A binding sites (nucleotides −962 to −957 and − 780 to −775) and three WRKY binding sites (nucleotides −777 to −772, −714 to −709, and −608 to −603) (Fig. S2). To elucidate the regulatory significance of these elements on *OsNF‐YC4* transcription, we conducted PCR‐based site‐directed mutagenesis, introducing deletions in the promoter region (D1, D2, D3). Each mutant promoter was subsequently cloned into a luciferase expression vector (Fig. 2a), and transiently expressed in leaves of *N. benthamiana*, a close wild relative of tobacco.

*Nicotiana benthamiana*, widely used for transient expression for genes from both monocots and dicots (Norkunas *et al*., 2018) was chosen as the host for these experiments due to the highly conserved nature of WRKY and RAV1A transcription factors and their binding sites across both monocot and dicot plant species. Previous studies have demonstrated that WRKY and RAV1A transcription factors in *N. benthamiana* can effectively target conserved motifs such as CAACA and TCACT, which are analogous to those found in rice and soybean promoters (Adachi *et al*., 2015; Mandal *et al*., 2023). This conservation suggests that the regulatory elements within the *OsNF‐YC4* promoter would interact similarly in *N. benthamiana*, making it a suitable system for heterologous expression studies.

The luciferase assays revealed that deletion of the second and third WRKY binding sites (D3) led to a 6.2‐fold increase (*P* < 0.01) in luciferase (LUC) activity, indicating that these sites may play a critical role in repressing *OsNF‐YC4* transcription. By contrast, deletions in the D1 and D2 regions, which targeted the first WRKY binding site and the adjacent RAV1A site, did not show obvious deviation in luciferase activity from the WT promoter (Fig. 2b). These findings suggest that the second and/or third WRKY binding sites may play a pivotal role in regulating *OsNF‐YC4* transcription, and their removal can enhance promoter activity, leading to increased gene expression.

In the rice genome, four RAV repressors have been identified: Os01g0140700 (OsRAV1), Os01g0141000 (OsRAV2), Os01g0693400 (OsRAV3), and Os05g0549800 (OsRAV4). Among these, OsRAV3 and OsRAV4 exhibit similarity to AtRAV1 (Steffens & Sauter, 2009; Rashid *et al*., 2012; Mohanty, 2021). Additionally, OsWRKY71 (Os02g0181300) and SUSIBA2 (SUgar SIgnaling in BArley 2) are known repressors targeting the W‐box motif (AGTCA/TGACT) (Sun *et al*., 2003; Zhang *et al*., 2004; Liu *et al*., 2007; Chujo *et al*., 2008; Kim *et al*., 2016; Jin *et al*., 2017; Zhou & Tang, 2019). To assess the binding capabilities of these RAVs/WRKYs to the predicted elements, we cloned and expressed OsRAV3, OsWRKY71, and OsWRKY121 (Os03g0741400, homologous to SUSIBA2) in *E. coli*. We designed seven fragments (P1 through P5, 198‐bp, and 193‐bp) of the *OsNF‐YC4* promoter (Figs 2c, S4) containing various combinations of W‐box motifs, labeled them with biotin, and performed electrophoretic mobility shift assays (EMSAs).

OsWRKY121 exhibited binding to the second W‐box (in P2 and P5), but not to the first (in P1) or third W‐box (in P3) motifs (Fig. 2d). Competitive assays using non‐biotin‐labeled fragments confirmed the specificity of OsWRKY121 for binding to the second W‐box motif (in P2) within the *OsNF‐YC4* promoter (Fig. 2e). Further assays using a 198‐bp fragment containing all three W‐box motifs and a synthesized 193‐bp fragment lacking the second W‐box demonstrated the preference of OsWRKY121 for binding to the full‐length fragment via the second W‐box (Fig. 2f).

OsWRKY71 exhibited binding to fragments containing the first and second W‐box motifs (in P1, P2, and P5), but not the third W‐box motif (in P3) or the negative control P4 (containing no W‐box) (Fig. S5a,b). Competitive assays with unlabeled probes confirmed the specificity of OsWRKY71 for the first and second W‐box regions (Fig. S5c). Deletion of the first or second W‐box motifs in synthesized fragments abolished binding with OsWRKY71 (Fig. S5d), further affirming the importance of these motifs for its binding.

Assays using OsRAV3 indicated its ability to bind both RAV1A motifs in the *OsNF‐YC4* promoter (Fig. S6). These findings collectively suggest that rice RAV and WRKY proteins can indeed bind to the corresponding motifs in the *OsNF‐YC4* promoter.

**Fig. 3:**
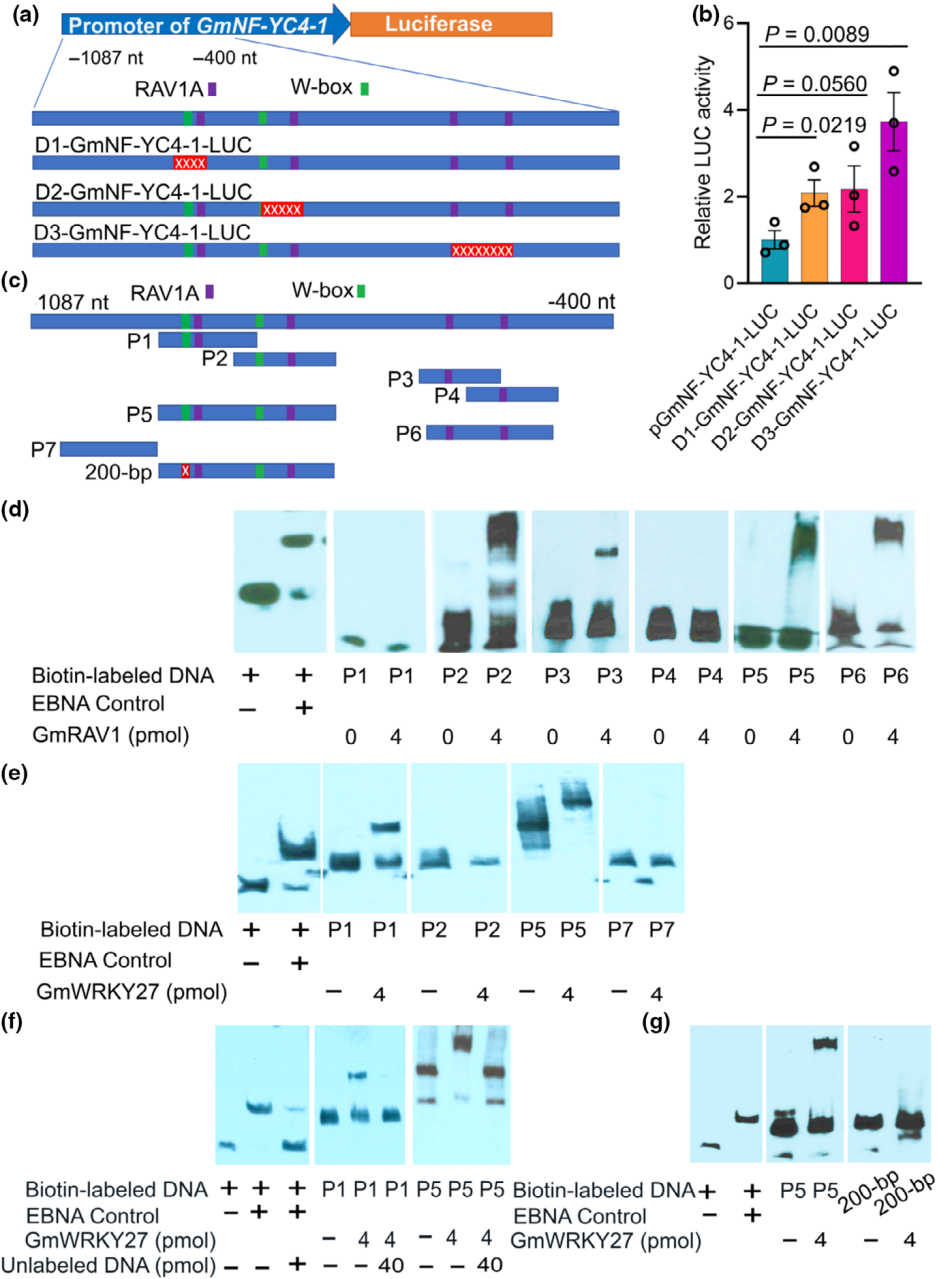
WRKY and RAV1A motifs in the *GmNF‐YC4‐1* promoter were bound by GmWRKY27 and GmRAV1, and their disruption increased *GmNF‐YC4‐1* transcription. (a) Structure of the *GmNF‐YC4‐*1 promoter and its deletion derivatives used in luciferase assays. The RAV1A (CAACA/TGTTG) and the WRKY (RGTCA/TGACY) binding sites are indicated as purple and green boxes, respectively. Deleted regions are indicated as red boxes with a string of ‘x's. (b) Relative luciferase activity (normalized against total protein content) was increased by all three deletions (D1, D2, D3). The promoters were fused to the luciferase reporter gene and transiently expressed in tobacco leaves after infiltration. Bar graphs show mean values ± error bars indicating the SE of mean, *n* = 3. Student's *t*‐test was used to compare the LUC (luciferase) activity between deletions and controls. (c) Structures of *GmNF‐YC4‐1* promoter fragments (P1–P7, 200‐bp), designed with different combinations of each RAV and W‐box motif. These promoter fragments were PCR‐amplified from WT plants and used for biotin labeling and EMSA (Electrophoretic Mobility Shift Assay) in (d–f). (d) EMSA showed that GmRAV1 could bind to the second RAV1A motif (in P2) and the third RAV1A motif (in P3) of the *GmNF‐YC4‐1* promoter, but not to the first RAV1A motif (in P1) or the fourth RAV1A motif (in P4). GmRAV1 was also able to bind to P5, which contained the first two RAV1A motifs, and P6, which contained the last two RAV1A motifs. (e) GmWRKY27 bound to P1 (1^st^ W‐box) and P5 (both W‐box motifs), but not P2 (2^nd^ W‐box). (f) Competition with unlabeled fragments confirmed that GmWRKY27 could specifically bind to the first W‐box (P1) motif in the *GmNF‐YC4‐1* promoter. (g) GmWRKY27 bound to P5, but not to a 200‐bp synthesized fragment lacking the first 5‐bp W‐box, with a 4‐bp N‐terminal deletion and a 3‐bp C‐terminal deletion of P5 (shown as a red box in c). This confirmed that the first W‐box was important for GmWRKY27 binding. Twenty femtomole of biotin‐labeled DNA fragments were used in (d–g). EBNA (Epstein–Barr nuclear antigen) from the EMSA kit was used as a positive control in (d–g).

To study the potential regulatory roles of RAVs and WRKYs in soybean, we investigated the *GmNF‐YC4‐1* promoter, revealing four RAV1A binding sites (nucleotides −891 to −887, −781 to −777, −592 to −588, and −526 to −522) and two WRKY binding sites (nucleotides −903 to −899 and −817 to −813) (Fig. 3a). To evaluate their regulatory significance, we introduced three deletions into the *GmNF‐YC4‐1* promoter (D1, D2, D3) (Figs 3a, S3). D1 removed the first WRKY and first RAV1A binding sites, D2 eliminated the second RAV1A site and a significant portion of the second WRKY binding site, while D3 excised nearly all the third RAV1A site and the entirety of the fourth RAV1A site (Fig. 3a). Subsequent luciferase assays conducted through transient expression in *N. benthamiana* revealed a 2.1‐fold increase in LUC activity for D1, a 2.2‐fold increase for D2, and a notable 3.7‐fold increase for D3 (Fig. 3b), indicating that disruption of the RAV1A and W‐box promoter elements may significantly enhance promoter activity, potentially increasing *GmNF‐YC4‐1* transcript levels *in vivo*.

GmRAV1 (Glyma.01G087500), analogous to AtRAV1, functions as a repressor by binding to the RAV1A motif (Zhang *et al*., 2010; K. Zhang *et al*., 2019). Similarly, GmWRKY27 (Glyma.03g224700) has been identified as a repressor through binding to the W‐box motif (Rushton *et al*., 2010; Wang *et al*., 2015). Recombinant GmRAV1 and GmWRKY27 proteins were produced using *E. coli*. To elucidate their binding capacity, we designed *GmNF‐YC4‐1* promoter fragments (Figs 3c, S7, S8) featuring diverse configurations of RAV1A and W‐box motifs for EMSAs with the purified GmRAV1 and GmWRKY27 proteins.

GmRAV1 demonstrated binding affinity to P2 (containing the second RAV1A motif), P3 (containing the third RAV1A), P5 (containing both the first and second RAV1A), and P6 (containing the third and fourth RAV1A motifs) (Fig. 3d). Notably, it did not bind to P1 (containing only the first RAV1A) nor P4 (containing only the fourth RAV1A motif) (Fig. 3d), indicating specific binding to the second and third RAV1A motifs. Synthesized fragments with the second RAV1A deleted (P2‐dRAV2^nd^), P3‐dRAV3^rd^ (with the 3^rd^ RAV1A deleted), and P8‐dRAV2^nd^ and 3^rd^ (with both 2^nd^ and 3^rd^ RAV1A deleted) showed a loss of binding with GmRAV1 (Fig. S8), confirming the significance of the second and third RAV1A motifs for GmRAV1 binding.

Fragments containing WRKY binding sites were also tested. GmWRKY27 exhibited binding to P1 (containing the first W‐box) and P5 (containing both the first and second W‐box), but not to P2 (containing only the second W‐box) or P7 (lacking any motif as a negative control) (Fig. 3e). This indicated specific binding of GmWRKY27 to the first W‐box. Competitive assays using non‐biotin‐labeled fragments confirmed the specificity of GmWRKY27 for the first W‐box motif (P1 and P5 fragments) in the *GmNF‐YC4‐1* promoter (Fig. 3f). Additional validation was achieved using a synthesized 200‐bp fragment (with 4‐bp N‐terminal deletion and 3‐bp C‐terminal deletion of P5, thus lacking the first W‐box) and a P1 fragment without the first W‐box (P1‐dWBOX1) (Fig. S8a). Results showed binding of GmWRKY27 to the P5 fragment but not to the 200‐bp fragment lacking the first W‐box (Fig. 3g), nor to P1‐dWBOX1 and P5‐dWBOX1, lacking the first W‐box (Fig. S8b). These experiments revealed the ability of RAV1 and WRKY transcription factors from *G. max* to bind to the *GmNF‐YC4‐*1 promoter. The fact that results were consistent between the dicotyledonous legume soybean and the monocotyledonous rice underscored the ability of RAV1 and WRKY transcriptional repressors to regulate the *NF‐YC4* promoter and suggest their roles in regulating the transcription of *NF‐YC4 in vivo*.

### Targeted deletion of repressor motifs in *NF‐YC4* promoters via CRISPR/Cas9 enhances gene expression, increases protein content and reduces starch in gene‐edited plants

Based on the results from both luciferase assays using transient expression in *N. benthamiana* and the EMSA analysis, we designed two sgRNAs (single guide RNAs) targeting specific regions within the *OsNF‐YC4* promoter to generate gene‐edited, transgene‐free rice plants. Employing *Agrobacterium*‐mediated transformation, we introduced the Cas9 and sgRNA expression cassettes into rice (Figs S9, S10). Out of 353 T0 events, 14 exhibited deletions in the *OsNF‐YC4* promoter. To identify transgene‐free CRISPR/Cas9 genome‐edited progeny, we monitored T‐DNA insertion via PCR, and tracked promoter deletions using both PCR and DNA sequencing. Screening of 219 T1 progeny from these 14 T0 plants enabled the identification of 97 heterozygous plants and 57 homozygous plants with target site deletions and 85 plants lacking deletions.

To ascertain the stable inheritance of deletions and if the deletions resulted in any higher protein or lower starch phenotype, we assessed T2 rice lines grown in both controlled growth chamber conditions and field settings. Notably, all T2 progeny from homozygous T1 plants showed homozygosity for promoter deletions through DNA sequencing. This robust stability underscores the effectiveness of CRISPR/Cas9‐mediated promoter editing in achieving desired genotypic traits in subsequent generations of rice plants.

**Fig. 4:**
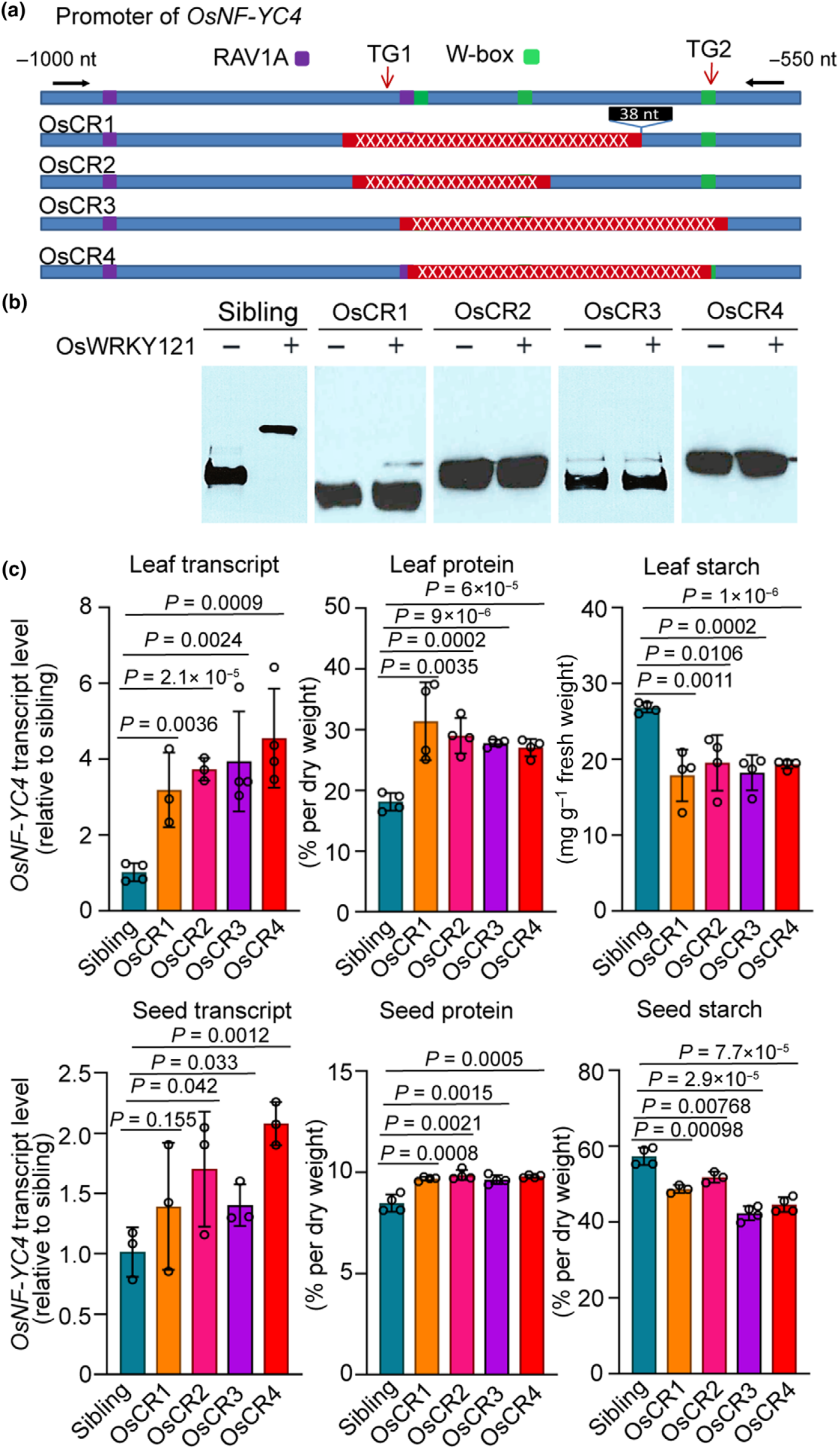
Targeted deletion of the RAV1A/W‐box motifs from the *OsNF‐YC4* promoter increased *OsNF‐YC4* transcript level and protein content in gene‐edited rice. (a) Structures of promoters in four gene‐edited CRISPR events with different deletions. Red rectangles with ‘x's represent homozygous deletions. Black arrows indicate the locations of the primers used for PCR amplification and biotin labeling for the EMSA (Electrophoretic Mobility Shift Assay) experiment in (b). TG1 and TG2 indicate two CRISPR/Cas9 cleavage sites designed to edit the *OsNF‐YC4* promoter. (b) EMSA demonstrated that OsWRKY121 lost binding affinity to the *OsNF‐YC4* promoter from the CRISPR/Cas9‐edited plants when the second W‐box was deleted. Biotin‐labeled DNA fragments (20 fmol) and OsWRKY121 (4 pmol) were used in the experiment. (c) In the CRISPR/Cas9‐edited plants, *OsNF‐YC4* transcript level was increased, relative to sibling, the value of which was set to 1. T3 leaves from gene‐edited plants showed elevated protein levels and decreased starch levels, and T4 seeds from these plants exhibited increased protein levels when compared to WT siblings. All values represent mean values ± error bars indicating the SE of mean, *n* ≥ 3 individual plants. Student's *t*‐test was used to compare the transcript levels, protein and starch contents of *OsNF‐YC4‐promoter‐*edited plants with controls.

We identified four distinct deletions in the *OsNF‐YC4* promoter: OsCR1 (missing nucleotides −814 to −696), OsCR2 (missing nucleotides −780 to −603), OsCR3 (missing nucleotides −777 to −606), and OsCR4 (missing nucleotides −820 to −649) (Figs 4a, S11). To assess the *in vivo* impact of the deletions on the RAV1A and WRKY binding sites, we conducted EMSAs using OsRAV3, OsWRKY121, and OsWRKY71 purified from *E. coli* along with various biotin‐labeled fragments of *OsNF‐YC4* promoters amplified by PCR from genomic DNA isolated from homozygous deletion lines and WT siblings. It was observed that OsRAV3, OsWRKY121, and OsWRKY71 either lost their binding ability or exhibited significantly reduced binding to all four deletions compared to the WT promoter fragments (Figs 4b, S12).

The quantitative real‐time polymerase chain reaction (RT‐qPCR) results revealed that the OsCR1 and OsCR2 deletions, which eliminated the second RAV1A and the first two WRKY binding elements, led to a 2.5‐ and 4‐fold increase in *OsNF‐YC4* transcript levels in rice leaves, respectively (Fig. 4a,c). Meanwhile, the OsCR3 and OsCR4 deletions, removing the second RAV1A and all three WRKY binding elements, showed over 4‐fold increases in *OsNF‐YC4* transcript levels in rice leaves (Fig. 4a,c). These lines exhibited a significant increase in leaf protein content (ranging from 43% to 68%, *P* < 0.01) and a decrease in leaf starch content (ranging from 30% to 41%, *P* < 0.05) compared to WT siblings (Figs 4c, S13a,b,e).

In addition, T3 rice seed harvested from all four types of deletion lines displayed a slight increase in transcript levels compared to WT siblings (Fig. 4c **)**. Furthermore, these transgene‐free seeds exhibited increased protein content, by *c.* 13–14% in OsCR1 lines, 12–17% in OsCR2 lines, 11–14% in OsCR3 lines and 14–15% in OsCR4 lines. Across these CRISPR‐edited‐promoter lines, elevated *OsNF‐YC4* transcript levels were accompanied by increased leaf and seed protein content and reduced leaf and seed starch content (Figs 4c, S13a,b,e). Notably, no discernible differences were observed in plant size or height, panicle features, seed size, seed weight, or seed yield per plant between these edited rice plants and WT‐sibling plants (*P* > 0.1) (Fig. S13c,d,f).

**Fig. 5:**
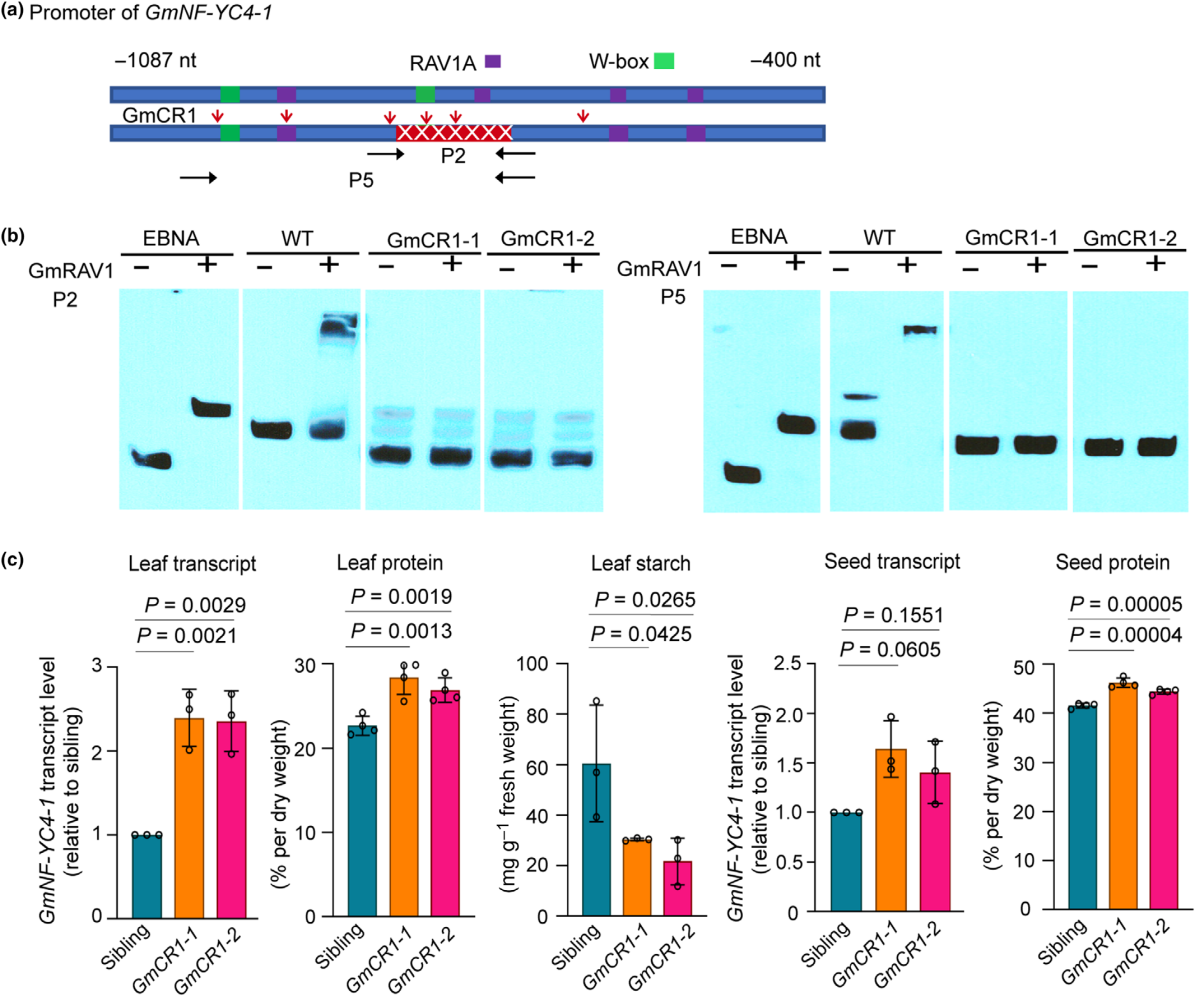
Targeted deletion of RAV1A/WRKY motifs from the *GmNF‐YC4‐1* promoter increased *GmNF‐YC4‐1* transcript level and protein content in gene‐edited soybeans. (a) Structure of the promoter in the identified CRISPR event harboring deletions. Red rectangle with ‘x's indicates homozygous deletion. Black arrows indicate the primer locations for PCR and biotin labeling of DNA fragments used in EMSA (Electrophoretic Mobility Shift Assay) experiments in (b). Red arrows denote designed Cas9 cut sites. (b) EMSA demonstrated that GmRAV1 lost binding affinity for the *GmNF‐YC4‐1* promoter from the CRISPR/Cas9‐edited plants when the second RAV1A motif was deleted. Biotin‐labeled DNA fragments (20 fmol) and GmRAV1 (4 pmol) were used in this experiment. (c) Promoter deletions led to increased *GmNF‐YC4‐1* transcript levels in gene‐edited soybean plants relative to siblings, the value of which was set to 1. T3 leaves from gene‐edited plants showed elevated protein levels and decreased starch levels. T4 Seeds from these plants exhibited increased protein levels. All data in bar chart represent mean values ± error bars indicating the SE of mean, *n* ≥ 3. Student's *t*‐test was used to compare the transcript levels, protein and starch contents of *GmNF‐YC4‐1‐promoter‐*edited plants with unedited Williams 82 sibling controls.

We designed six sgRNAs targeting specific regions within the *GmNF‐YC4‐1* promoter (Figs 5a, S14, S15). T2 soybean plants lacking integrated gene editing constructs were grown in the field, and their genotypes were determined via PCR and sequencing. Two CRISPR lines were identified based on their deletion profiles at the RAV1A and W‐box motifs. Both lines exhibited a homozygous 55‐nt deletion from −825 to −770, effectively eliminating the second RAV1A site and the second W‐box site (GmCR1‐1, 1‐2) (Figs 5a, S16). Given the importance of the second RAV1A site for GmRAV1 binding (as demonstrated in Fig. 3), we assessed the binding ability of purified GmRAV1 proteins to the promoter regions P2 and P5, using biotin‐labeled promoter fragments amplified from genomic DNA isolated from homozygous deletion lines and WT siblings (Fig. 5b). The PCR‐amplified promoter regions from both the GmCR1‐1 and GmCR1‐2 lines exhibited a loss of GmRAV1 binding (Fig. 5b).

To further validate the impact of CRISPR deletions on *GmNF‐YC4‐1*, T3 soybean plants were cultivated in the field and evaluated for associated traits. Comparable analysis with WT siblings revealed a marked increase in *GmNF‐YC4‐1* transcript levels (*c.* 2‐fold) in soybean leaves for both deletion lines (Fig. 5c). Moreover, these lines exhibited a significant reduction in leaf starch content (49–64% decrease, *P* < 0.01) and a concurrent increase in leaf protein content (18–25% increase, *P* < 0.01) in T3 plants compared to controls (Fig. 5c). Furthermore, these edited soybean lines demonstrated elevated seed protein content (6–11% increase, *P* < 0.01) compared to control (Fig. 5c), although no significant difference was observed in seed *GmNF‐YC4‐1* transcript levels compared to siblings (*P* > 0.05). The starch content of mature soybean seeds was not measured or included in Fig. 5 because it is already very low and not expected to show significant variation. Notably, no discernible variations were detected in plant/seed growth and development, in plant biomass, and seed weight per seed or per plant between the CRISPR/Cas9‐edited soybeans and their WT siblings (*P* > 0.05) (Fig. S17).

To explore the expression pattern of *AtNF‐YC4*, *OsNF‐YC4* and *GmNF‐YC4‐1*, we conducted an analysis using public online databases. It was observed that these three genes are primarily expressed in leaf, hypocotyl, pollen, and silique tissues, with lower expression in young seeds and almost negligible expression in mature seeds (Fig. S18).

## Discussion

The increasing incidence of diseases such as cancer and type‐2 diabetes has triggered a shift towards low‐carbohydrate, high‐protein diets, which are valued for their preventive and therapeutic benefits. These diets aid in weight loss, muscle gain, and overall health improvement (Berkowitz, 2000). However, excessive protein intake can have adverse effects, including heart, kidney and liver issues (Berkowitz, 2000). To mitigate these risks, the American Heart Association recommends a balanced diet comprising 10–35% of daily calories from protein, 45–65% from carbohydrates, and 20–35% from fat.

Despite these recommendations, a significant portion of the global population relies heavily on starchy staple foods and plant‐based protein sources. For instance, rice is a protein‐poor crop, with only *c.* 10% protein content in its grains (Jiang *et al*., 2016). Diets can be improved by including legumes, such as soybeans, which contain higher protein levels in their seeds. Consequently, there is an urgent need to enhance protein levels in rice and soybean within reasonable bounds (Krishnan *et al*., 2007; Peng *et al*., 2014; Jiang *et al*., 2016). Such efforts are imperative for addressing nutritional deficiencies and promoting well‐being. While transgenic methods have been employed to improve crops (Bailey‐Serres *et al*., 2019; Kotula *et al*., 2020), genome editing offers a transgene‐free alternative through gene knockout (Li *et al*., 2012, 2017; Qi *et al*., 2013; Gilbert *et al*., 2014; Shan *et al*., 2014; Chavez *et al*., 2015, 2016; Konermann *et al*., 2015; Hu *et al*., 2018; Kwon *et al*., 2020; Lin *et al*., 2020; Lu *et al*., 2020). Mutagenesis of *cis*‐regulatory elements through promoter mutagenesis has enabled quantitative variation in tomato and maize (Tanimura *et al*., 2016; Rodríguez‐Leal *et al*., 2017; Y. Zhang *et al*., 2019; Li *et al*., 2020; Liu *et al*., 2021). However, the utilization of *cis*‐element modification to upregulate gene expression by removing repressor‐binding motifs remains relatively unexplored.

Previous studies have shown that CRISPR/Cas9‐mediated gene editing can be employed not only for knockout but also for generating overexpression alleles. Two notable approaches in this context involve inducing chromosomal inversions and targeting upstream open reading frames (uORFs) in the 5′‐untranslated regions (5′‐UTR) of genes. Čermák *et al*. (2017) developed a toolkit enabling advanced genome engineering, including chromosomal inversions that reposition regulatory elements like enhancers to boost gene expression. Zhang *et al*. (2018) demonstrated that genome editing of uORFs in plant genes could control translation, effectively increasing of gene expression. These studies collectively highlight the diverse approaches that CRISPR/Cas9 can be used to enhance gene expression in plants, providing a strong foundation for our own work on targeting noncoding regulatory motifs in rice and soybean *NF‐YC4* promoters.

In our study, we employed genome editing to remove repressor‐binding sites in the *NF‐YC4* promoters in rice and soybean. Editing of the *NF‐YC4* promoter resulted in decreased repressor binding and increased *NF‐YC4* expression. This, in turn, led to elevated protein and reduced carbohydrate levels in both crops. These results highlighted the potential of targeted *cis*‐element modification as a novel strategy for crop improvement, offering valuable insights into enhancing agronomic traits without transgenic approaches.

The deletion of the second RAV1A and second W‐box motifs in the *OsNF‐YC4* promoter elevated *OsNF‐YC4* expression in rice, impacting protein and carbohydrate composition. We hypothesized that the second W‐box may be important in regulating *OsNF‐YC4* transcript levels, supported by the binding of two WRKY transcription factors, namely OsWRKY71 and OsWRKY121 (Figs 2, 4, S5). Similarly, the deletion of regions encompassing the second RAV1A motif in the *GmNF‐YC4‐1* promoter led to increased *GmNF‐YC4‐1* expression and elevated protein levels in the leaves and seeds. We postulated that the second and third RAV1A sites might be crucial in regulating *GmNF‐YC4‐1* expression, supported by the high‐luciferase activity upon their deletion and the binding of this region by both GmRAV1 and GmWRKY27 (Fig. 3).

The transcript levels of both *GmNF‐YC4‐1* and *OsNF‐YC4* in the seeds of the CRISPR‐edited plants were similar to or only marginally different from those in the WT‐sibling plants, unlike the significant increase observed in leaf tissues (2–4 times) (Figs 4c, 5c). Despite lesser changes in transcript levels, the seed protein content in the CRISPR‐edited plants increased by 11–17% in rice (Fig. 4c) and 6–11% in soybean (Fig. 5c), consistent with the enhancements observed in seeds of *OsNF‐YC4‐OE* (Fig. 1f; 18–21%) or *GmNF‐YC4‐1‐OE* (8–11%) (O'Conner *et al*., 2018). Expression pattern analysis of *AtNF‐YC4*, *OsNF‐YC4*, and *GmNF‐YC4‐1* in transcriptomes revealed predominant expression in leaf, pollen, and siliques, with lower expression in young seeds and minimal expression in mature seeds (Fig. S18). This suggests that the crucial role of OsNF‐YC4/GmNF‐YC4‐1 for seed protein content may be in source organs, potentially involving nitrogen mobilization from leaves to seeds rather than direct protein storage in seeds.

The bioinformatic analysis (Table S2) suggests a possible conservation of *NF‐YC4* repression by RAV1 and/or WRKY across monocots and dicots. This, coupled with our demonstrated success in increasing crop protein content through targeted deletions in *NF‐YC4* promoters using gene‐editing tools, suggests a universal mechanism for enhancing crop protein content using tools such as meganuclease, TALENS and CRISPR/Cas gene editing systems.

In conclusion, our study illustrates the use of SDN‐1 to upregulate endogenous genes, which is an adaptable method for the generation of targeted promoter deletions. Here, editing of *cis*‐repressor elements in a promoter increased gene expression and created gain‐of‐function phenotypes without transgenes. Future studies can test the generalizability of this method in editing other types of *cis*‐elements for targeted expression changes. This research paves the way for future applications that aim to enhance crop productivity and nutritional quality through precise genome editing.

## Competing interests

LL is an inventor on several patents related to the work described in this manuscript. The authors declare no other competing interests.

## Author contributions

LL designed the study. LW, SOC, RT, WZ, SC and KT performed experiments. LW, SOC, LL, EEE, HB, BY and DFV analyzed data. LW and LL prepared the manuscript. All authors have revised and approved the final version of the manuscript. LW and SOC contributed equally to this work.

## Supporting information


**Fig. S1** Comparison of *OsNF‐YC4‐OE* seeds and WT‐sibling seeds.
**Fig. S2** The location of predicted RAV1A and WRKY binding motifs in the promoter of *OsNF‐YC4*.
**Fig. S3** The locations of predicted RAV1A and WRKY binding motifs in the promoter of *GmNF‐YC4‐1*.
**Fig. S4** Sequence details of fragments within the *OsNF‐YC4* promoter utilized for EMSA.
**Fig. S5** The WRKY family member OsWRKY71 bound to the first and the second WRKY binding sites in the *OsNF‐YC4* promoter.
**Fig. S6** The RAV family member OsRAV3 binds to the first and second RAV1A binding motifs (TGTTG) in the *OsNF‐YC4* promoter.
**Fig. S7** Sequence information of fragments from the promoter of *GmNF‐YC4‐1* used for EMSAs.
**Fig. S8** GmRAV1 and GmWRKY27 bind to RAV1A and WRKY motifs in the promoter of *GmNF‐YC4‐1*.
**Fig. S9** Guide RNA design for the *OsNF‐YC4* promoter.
**Fig. S10** Map of the CRISPR/Cas9 construct prCas9‐gOsNF‐YC4 for editing the *OsNF‐YC4* gene promoter in rice.
**Fig. S11** Sequences of the *OsNF‐YC4* promoters in CRISPR‐edited rice plants.
**Fig. S12** OsWRKY71/OsRAV3 lost or significantly reduced binding to the promoter of *OsNF‐YC4* from CRISPR/Cas9‐edited plants when the first and second W‐box motifs and the second RAV1A motif were deleted.
**Fig. S13** Leaf starch content was decreased in otherwise morphologically similar CRISPR/Cas9‐edited rice plants with deletions in RAV1A/W‐box in the *OsNF‐YC4* promoter.
**Fig. S14** Guide RNAs designed for the *GmNF‐YC4‐1* promoter.
**Fig. S15** Map of the CRISPR/Cas9 construct prCas9‐gGmNF‐YC4‐1 for editing the *GmNF‐YC4‐1* promoter in soybean.
**Fig. S16** Sequences in the *GmNF‐YC4‐1* promoters in soybean CRISPR‐edited plants.
**Fig. S17** Morphological assessment of CRISPR/Cas9‐edited soybean plants with deletions in RAV1A/W‐box in the *GmNF‐YC4‐1* promoter, compared to WT siblings.
**Fig. S18** Expression pattern of *AtNF‐YC4*, *OsNF‐YC4* and *GmNF‐YC4‐1* from online public databases.
**Table S1** List of primers and their applications.
**Table S2** Bioinformatic analysis of multiple crop species revealed RAV and WRKY binding motifs were conserved in the promoters of the NF‐YC4 orthologs.
Please note: Wiley is not responsible for the content or functionality of any Supporting Information supplied by the authors. Any queries (other than missing material) should be directed to the *New Phytologist* Central Office.

## Data Availability

Data supporting the findings of this work are available in the main text and in the figures and tables in Supporting Information. The genetic materials generated and analyzed in this current study are available from the corresponding author upon request.
