# Supplementary material for: CRISPR/Cas9‐based editing of NF‐YC4 promoters yields high‐protein rice and soybean
Source: New Phytol. 2024 Sep 22;245(5):2103–16. doi: 10.1111/nph.20141 (PMC11798907; doi:10.1111/nph.20141)
Supplement: Supplementary file 1 — Fig. S1 Comparison of OsNF‐YC4‐OE seeds and WT‐sibling seeds. Fig. S2 The location of predicted RAV1A and WRKY binding motifs in the promoter of OsNF‐YC4. Fig. S3 The locations of predicted RAV1A and WRKY binding motifs in the promoter of GmNF‐YC4‐1. Fig. S4 Sequence details of fragments within the OsNF‐YC4 promoter utilized for EMSA. Fig. S5 The WRKY family member OsWRKY71 bound to the first and the second WRKY binding sites in the OsNF‐YC4 promoter. Fig. S6 The RAV family member OsRAV3 binds to the first and second RAV1A binding motifs (TGTTG) in the OsNF‐YC4 promoter. Fig. S7 Sequence information of fragments from the promoter of GmNF‐YC4‐1 used for EMSAs. Fig. S8 GmRAV1 and GmWRKY27 bind to RAV1A and WRKY motifs in the promoter of GmNF‐YC4‐1. Fig. S9 Guide RNA design for the OsNF‐YC4 promoter. Fig. S10 Map of the CRISPR/Cas9 construct prCas9‐gOsNF‐YC4 for editing the OsNF‐YC4 gene promoter in rice. Fig. S11 Sequences of the OsNF‐YC4 promoters in CRISPR‐edited rice plants. Fig. S12 OsWRKY71/OsRAV3 lost or significantly reduced binding to the promoter of OsNF‐YC4 from CRISPR/Cas9‐edited plants when the first and second W‐box motifs and the second RAV1A motif were deleted. Fig. S13 Leaf starch content was decreased in otherwise morphologically similar CRISPR/Cas9‐edited rice plants with deletions in RAV1A/W‐box in the OsNF‐YC4 promoter. Fig. S14 Guide RNAs designed for the GmNF‐YC4‐1 promoter. Fig. S15 Map of the CRISPR/Cas9 construct prCas9‐gGmNF‐YC4‐1 for editing the GmNF‐YC4‐1 promoter in soybean. Fig. S16 Sequences in the GmNF‐YC4‐1 promoters in soybean CRISPR‐edited plants. Fig. S17 Morphological assessment of CRISPR/Cas9‐edited soybean plants with deletions in RAV1A/W‐box in the GmNF‐YC4‐1 promoter, compared to WT siblings. Fig. S18 Expression pattern of AtNF‐YC4, OsNF‐YC4 and GmNF‐YC4‐1 from online public databases. Table S1 List of primers and their applications. Table S2 Bioinformatic analysis of multiple crop species revealed RAV and WRKY binding motifs w [file NPH-245-2103-s001.pdf]

New Phytologist

***New Phytologist* Supporting Information**

Article title: **CRISPR/Cas9-based editing of *NF-YC4* promoters yields high-protein rice and soybean**

Authors: **Lei Wang, Seth O'Conner, Rezwan Tanvir, Wenguang Zheng, Samuel Cothron, Katherine Towery, Honghao Bi, Evan E. Ellison, Bing Yang, Daniel F. Voytas and Ling Li**

Article acceptance date: 28 August 2024

The following Supporting Information is available for this article:

Fig. S1 Comparison of rice *OsNF-YC4-OE* seeds and WT-sibling seeds.

Fig. S2 The location of predicted RAV1A and WRKY binding motifs in the promoter of *OsNF-YC4*.

Fig. S3 The locations of predicted RAV1A and WRKY binding motifs in the promoter of *GmNF-YC4-1*.

Fig. S4 Sequence details of fragments within the *OsNF-YC4* promoter utilized for EMSA (Electrophoretic Mobility Shift Assay).

Fig. S5 The WRKY family member OsWRKY71 bound to the first and the second WRKY binding sites in the *OsNF-YC4* promoter.

Fig. S6 The RAV family member OsRAV3 binds to the first and second RAV1A binding motifs (TGTTG) in the *OsNF-YC4* promoter.

Fig. S7 Sequence information of fragments from the promoter of *GmNF-YC4-1* used for EMSAs (Electrophoretic Mobility Shift Assay).

Fig. S8 GmRAV1 and GmWRKY27 bind to RAV1A and WRKY motifs in the promoter of *GmNF-YC4-1*.

Fig. S9 Guide RNA design for the *OsNF-YC4* promoter.

Fig. S10 Map of the CRISPR/Cas9 construct prCas9-gOsNF-YC4 for editing the *OsNF-YC4* gene promoter in rice.

Fig. S11 Sequences of the *OsNF-YC4* promoters in CRISPR-edited rice plants.

Fig. S12 OsWRKY71/OsRAV3 lost or significantly reduced binding to the promoter of *OsNF-YC4* from CRISPR/Cas9-edited plants when the first and second W-box motifs and the second RAV1A motif were deleted.

Fig. S13 Leaf starch content was decreased in otherwise morphologically similar CRISPR/Cas9-edited rice plants with deletions in RAV1A/ W-box in the *OsNF-YC4* promoter.

Fig. S14 Guide RNAs designed for the *GmNF-YC4-1* promoter.

Fig. S15 Map of the CRISPR/Cas9 construct prCas9-gGmNF-YC4-1 for editing the *GmNF-YC4-1* promoter in soybean.

Fig. S16 Sequences in the *GmNF-YC4-1* promoters in soybean CRISPR-edited plants.

Fig. S17 Morphological assessment of CRISPR/Cas9-edited soybean plants with deletions in RAV1A/ W-box in the *GmNF-YC4-1* promoter, compared to WT siblings.

Fig. S18 Expression pattern of *AtNF-YC4*, *OsNF-YC4* and *GmNF-YC4-1* from online public databases.

Table S1 List of primers and their applications.

Table S2 Bioinformatic analysis of multiple crop species revealed RAV and WRKY binding motifs were conserved in the promoters of the NF-YC4 orthologs.

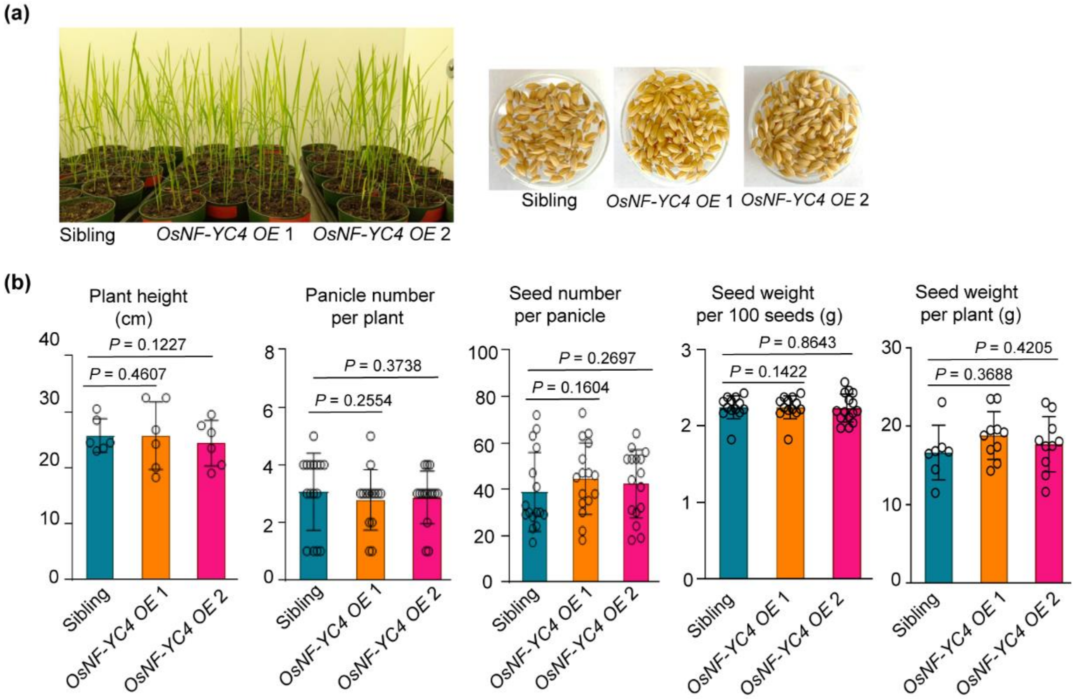

Fig. S1 Comparison of rice *OsNF-YC4-OE* seeds and WT-sibling seeds. (a) Phenotype of *OsNF-YC4-OE* plants and seeds. (b) Evaluation of plant height, panicle number per plant, seed number per panicle, and seed weight per 100 seeds or per plant to assess the effect of *OsNF-YC4-OE* plants compared to sibling controls. The conditions were 28 ºC in the light and 25 ºC in the dark, with a long-day photoperiod of 16-h light and 8-h dark in growth chamber. Data were recorded for the T3 generation seeds. All values in bar charts represent mean values ± error bars indicating the standard error of mean. $n \geq 7$ plants, three biological replicates. Student's *t*-test was used to compare *OsNF-YC4-OE* rice plants to controls; $P > 0.05$ for these tests.

ACTTAATTAACTAGTTGAGATCGACATGCATGGATGCGTGAATCTAT**TGTTG**CATTTTTTCTAATGGATAGTTAG
ATTTGTTCCAGCTTTTTTTTTGTCAATTCTGTTACATTTGCAGTGTCTTCAACTTCTGCATCATTCTTCTCTATT
CTTCCTTCACTTTCTTTCTTGAACTAGAAAAGCAAACCAGAGTTCTTTTTCCCCCTCTTCTACATAAACGAAAAC
AGCT**TGTTGACT**GGCTCCCTAGAGCTTTTTGTAAGTTGATCATCGAAGTAGCTAGTTCTCTTCACTTATC**AGTCA**
TCACTGTTCTATGTTCTATCTGCATTTTCCTTGATTTTGTACTTTTCCTGAACGAAAGGACAATCCTTAGCCATC
ATAATGCTATGATCGACTTATTCTGA**AGTCA**TCCGGCCTCGATCCTCTTTTGTTTCGGTGAGATCTGTAATGGTT
TAGGAAAATTATGGATCTCTGAAAAATAAGAAACATCTAGTAGTATATGAAATTAAGAAATGTCGGACCACGTCA
AATCCTACTGGTATCATATACCAGATAACTAACTTTTTGAATGGAACCAAACACGAGATTGTGAATATATAGCAG
TAAGAAATACAACCCCGAAGGGTAAAGGAGAAAAAGGAAAAGCAGTTTAGTGTACGAGCTGTAGGAGTAGCTTTG
CTCCTGCCAAGGCCCAGATCCTTGCCTCTTCCTTCATTTCTGCGAGGAGACCAGGCACTGAGGTCTCATTGTGAT
CGAAGATTCTCCTGTTTCTCTCCTTCCAGATCTCCTTTGGGAACACCCTGTGTGTTTGCAAGCGCTTAGCTTCAT
TTTCATGCATGATATGATCTCTGGATAGAAGATAAGTAGCCCCCACACATGCACTTCGCCACCTATAGCCAGCCT
CGAGTTCTCATCTGTTTGATCCGTCGCACATGTGGAGCAACGAAGCTCTAGAACACAGCACACACAGCTACAAAT
CGACTGTAATTAAGGTACGTATATATAGGTGACA**ATG**GACAACCAGCAGCTACCCTACGCCGGTCAGCCGGCGGC
CGCAGGCGCCGGAGCCCCGGTGCCGGGCGTGCCTGGCGCGGGCGGGCCGCCGGCGGTGCCGCACCACCACCTGCT
CCAGCAGCAGCAGGCGCAGCTGCAGGCGTTCTGGGCGTACCAGCGGCAGGAGGCGGAGCGCGCGTCGGCGTCGGA
CTTCAAGAACCACCAGCTGCCGCTGGCGCGGATCAAGAAGATCATGAAGGCGGACGAGGACGTGCGCATGATCTC
GGCGGAGGCGCCCGTGCTGTTCGCCAAGGCGTGCGAGCTCTTCATCCTGGAGCTCACCATCCGCTCGTGGCTGCA
CGCCGAGGAGAACAAGCGCCGCACCCTGCAGCGCAACGACGTCGCCGCCGCCATCGCGCGCACCGACGTGTTCGA
CTTCCTCGTCGACATCGTGCCGCGGGAGGAGGCCAAGGAGGAGCCCGGCAGCGCGCTCGGGTTCGCGGCGGGAGG
GCCCGCCGGCGCCGTTGGAGCGGCCGGCCCCGCCGCGGGGCTGCCGTACTACTACCCGCCGATGGGGCAGCCGGC
GCCGATGATGCCGGCGTGGCATGTTCCGGCGTGGGACCCGGCGTGGCAGCAAGGAGCAGCGCCGGATGTGGACCA
GGGCGCCGCCGGCAGCTTCAGCGAGGAAGGGCAGCAAGGTTTTGCAGGCCATGGCGGTGCGGCAGCTAGCTTCCC
TCCTGCACCTCCAAGCTCCGAA**TAG**TGATGATCCATATGGTTCCATGCATGCATCGCTGAGGTGCTAGCTAGCTA

RAV1A (1st): -962 to -957 nt; **RAV1A** (2nd): -780 to -775 nt.
W-box (1st): -777 to -772 nt; **W-box** (2nd): -714 to -709 nt; **W-box** (3rd): -608 to -603 nt.

Fig. S2 The location of predicted RAV1A and WRKY binding motifs in the promoter of *OsNF-YC4*. The promoter contains two RAV1A target motifs, represented by "TGTTG/CAACA", and three WRKY target motifs, represented by "TGACT/AGTCA". RAV1A target motifs are in purple font, while WRKY target motifs are in green font. Additionally, the overlap between the RAV1A binding motif "TGTTG" and the WRKY binding motif "TGACT" (W-box) is underlined. The *OsNF-YC4* gene coding sequence is highlighted in yellow.

ACACAAAAGTGACAAAATGTGGACTTGAAAATCACATGCTAGAGCAAAGCAAGGTTCAGATGAAGCCAAATGGCTA
GAACGTAAATGCTGCAAAAATCAATTATATTTGAATCTCACGGGCATTGAACAAAAGAGAATGGACCCATCAATGG
GGCATTGACATGGATGGATGGAACACCCTCAT**AGTCA**CATGCCA**CAACA**GAGTAACGCTAAACAAAATGTATTAAC
AAAAACTATAGGATCATGCTTTCTTAATTTAAAAACATAGGT**GGTCA**GTTTTTGTTAACATTAATTTTTAGGATTA
TT**TGTTG**AAGACAAATATGGAAGGTTTATAATAATTTATGTGCATTACTCAATGTAATCCTTAAAATGAAAATAAC
TAAACTTTTTTTTTTTGGATAGTTAAACAACTACGAAGCTAAATGAATAATTAAATCAATATTAATTTTGATTAAA
AATAAGATTAATAATATCCCTAATATTGTGAACAAACTT**TGTTG**GTAATGTAAAAAAAATTAAAAGAAACAAGATT
AAATTACGTATTTAATAATTTAAGATTAA**TGTTG**TTTAATTTGATTTTTTAAATATTTATCTTTCTTTTGAATTTG
ATTCTTTAGTTCACTTAAGATTATGATCGTTAATAATTCTAAAAGATAAATGTCATCAATCTTGAATTGATTGAAA
GATTAAATTAAATAAAAAGAATTAAGATAAATGATGAAAATACATTTAAAAAATAAGATGAAACAAAATGTGTCAT
TTAACCAAAAAAAAAAAGGAAAAATAAATTAAAAAAAGGAACTTACCCTTCCCCTCAAAAAAGAAAAGAAAAAAAAA
AAGGAACTTACCCTTGGTTGTTCCGTTGAAATTGAAAAAACAAACCCTAAACTTACCTTAACCTAGGTCCTAGGGC
ACTGGTCGGATATGTTATTGTTTAGTTTACCTTATCCCACCACATACATAGTTTTTTTTTCTTAAATTTCCCAAT
CAATTCCATCCATCGGGTCTTTACTCTCTTACCCAACCCACACACACTCTTTCTCTCTCTCTCTTTCCCTGATCAT
CAAAATCAGAAAAAATTGGGGGA**ATG**GAGACCAACAACCAGCAACAACAACAACAAGGAGCTCAAGCCCAATCGGG
ACCCTACCCCGTCGCCGGCGCCGGCGGCAGTGCAGGTGCAGGTGCAGGCGCTCCTCCCCCTTTCCAGCACCTTCTC
CAGCAGCAGCAGCAGCAGCTCCAGATGTTCTGGTCTTACCAGCGTCAAGAAATCGAGCACGTGAACGACTTTAAGA
ATCACCAGCTCCCTCTTGCCCGCATCAAGAAGATCATGAAGGCCGACGAGGATGTCCGCATGATCTCCGCCGAGGC
CCCCATCCTCTTCGCCAAGGCCTGCGAGCTCTTCATCCTCGAGCTCACCATCCGCTCCTGGCTCCACGCCGAGGAG
AACAAGCGCCGCACCCTCCAGAAGAACGACATCGCCGCCGCCATCACCCGCACCGACATTTCGACTTCCTCGTTG
ATATTGTCCCCCGCGACGAGATCAAGGACGACGCTGCTCTTGTGGGGGCCACCGCCAGTGGGGTGCCTTACTACTA
CCCGCCCATTGGACAGCCTGCCGGGATGATGATTGGCCGCCCCGCCGTCGATCCCGCCACCGGGGTTTATGTCCAG
CCGCCCTCCCAGGCATGGCAGTCCGTCTGGCAGTCCGCTGCCGAGGACGCTTCCTATGGCACCGGCGGGGCCGGTG
CCCAGCGGAGCCTTGATGGCCAGAGT**TGA**

RAV1A (1st): -891 to -887 nt; **RAV1A** (2nd): -781 to -777 nt; **RAV1A** (3rd): -592 to -588 nt; **RAV1A** (4th): -526 to -522 nt.

W-box (1st): -817 to -813 nt; **W-box** (2nd): -817 to -813 nt.

Fig. S3 The locations of predicted RAV1A and WRKY binding motifs in the promoter of *GmNF-YC4-1*. The promoter contains four RAV1A target motifs, represented by “TGTTG/CAACA”, and two W-box motifs, represented by “TGACT/AGTCA”. RAV1A target motifs are in purple font, while WRKY target motifs are in green font. The *GmNF-YC4-1* gene coding sequence is highlighted in yellow.

OsNF-YC4 promoter:
TTTTGGATATCTGAAGCAGTAGAGCTCGTTCACTCAAACAGGCTAGCCTGTTTTGCTTTCTGG
TAAGAACTTAATTAACTAGTTGAGATCGACATGCATGGATGCGTGAATCTAT**TGTTG**CATTTT
TTCTAATGGATAGTTAGATTTGTTCCAGCTTTTTTTTTGTCAATTCTGTTACATTTGCAGTGT
CTTCAACTTCTGCATCATTCTTCTCTATTCTTCCTTCACTTTCTTTCTTGAACTAGAAAAGCA
AACCAGAGTTCTTTTTCCCCCTCTTCTACATAAACGAAAACAGCT**TGTTGACT**GGCTCCCTAG
AGCTTTTTGTAAGTTGATCATCGAAGTAGCTAGTTCTCTTCACTTATC**AGTCA**TCACTGTTCT
ATGTTCTATCTGCATTTTCCTTGATTTTGTACTTTTCCTGAACGAAAGGACAATCCTTAGCCA
TCATAATGCTATGATCGACTTATTCTGA**AGTCA**TCCGGCCTCGATCCTCTTTTGTTTCGGTGA
GATCTGTAATGGTTTAGGAAAATTATGGATCTCTGAAAAATAAGAAACATCTAGTAGTATATG
AAATTAAGAAATGTCGGACCACGTCAAATCCTACTGGTATCATATACCAGATAACTAACTTTT
TGAATGGAACCAAACACGAGATTGTGAATATATAGCAGTAAGAAATACAACCCCGAAGGGT

OsNF-YC4 promoter-P1:
ACCAGAGTTCTTTTTCCCCCTCTTCTACATAAACGAAAACAGCT**TGTTGACT**GGCTCCCTAGA
GCTTTTTGTAAGTTGATCATCGAAGTAGCT

OsNF-YC4 promoter-P2：
TGGCTCCCTAGAGCTTTTTGTAAGTTGATCATCGAAGTAGCTAGTTCTCTTCACTTATC**AGTC**
ATCACTGTTCTATGTTCTATCTGCATTTTCCTTGATTTTGTACTTTTCCTGAACGAAAGGACA
ATCCTT

OsNF-YC4 promoter-P3：
TCCTGAACGAAAGGACAATCCTTAGCCATCATAATGCTATGATCGACTTATTCTGA**AGTCA**TC
CGGCCTCGATCCTCTTTTGT

OsNF-YC4 promoter-P4：
TCTGCATTTTCCTTGATTTTGTACTTTTCCTGAACGAAAGGACAATCCTTAGCCATCATAATG
CT

OsNF-YC4 promoter-P5：
ACCAGAGTTCTTTTTCCCCCTCTTCTACATAAACGAAAACAGCT**TGTTGACT**GGCTCCCTAGA
GCTTTTTGTAAGTTGATCATCGAAGTAGCTAGTTCTCTTCACTTATC**AGTCA**TCACTGTTCTA
TGTTCTATCTGCATTTTCCTTGATTTTGTACTTTTCCTGAACGAAAGGACAATCCTTAGCCAT
CATAATGCTATGATCGACTTATTCTGA**AGTCA**TCCGGCCTCGATCCTCTTTTGT

OsNF-YC4 promoter-198-bp：
AACAGCT**TGTTGACT**GGCTCCCTAGAGCTTTTTGTAAGTTGATCATCGAAGTAGCTAGTTCTC
TTCACTTATC**AGTCA**TCACTGTTCTATGTTCTATCTGCATTTTCCTTGATTTTGTACTTTTCC
TGAACGAAAGGACAATCCTTAGCCATCATAATGCTATGATCGACTTATTCTGA**AGTCA**TCCGG
CCTCGATCCT

OsNF-YC4 promoter-193-bp：
AACAGCT**TGTTGACT**GGCTCCCTAGAGCTTTTTGTAAGTTGATCATCGAAGTAGCTAGTTCTC
TTCACTTATC~~**AGTCA**~~TCACTGTTCTATGTTCTATCTGCATTTTCCTTGATTTTGTACTTTTCC
TGAACGAAAGGACAATCCTTAGCCATCATAATGCTATGATCGACTTATTCTGA**AGTCA**TCCGG
CCTCGATCCT

Fig. S4 Sequence details of fragments within the *OsNF-YC4* promoter utilized for EMSA (Electrophoretic Mobility Shift Assay). The RAV1A target motif is denoted in purple font, while WRKY target motif is indicated in green font. The overlap between the RAV1A binding motif "TGTTG" and the WRKY binding motif "TGACT" is underlined. Struck-through "~~XXX~~" text in the *OsNF-YC4* promoter-193-bp denotes the 5-bp deletion corresponding to the second WRKY motif.

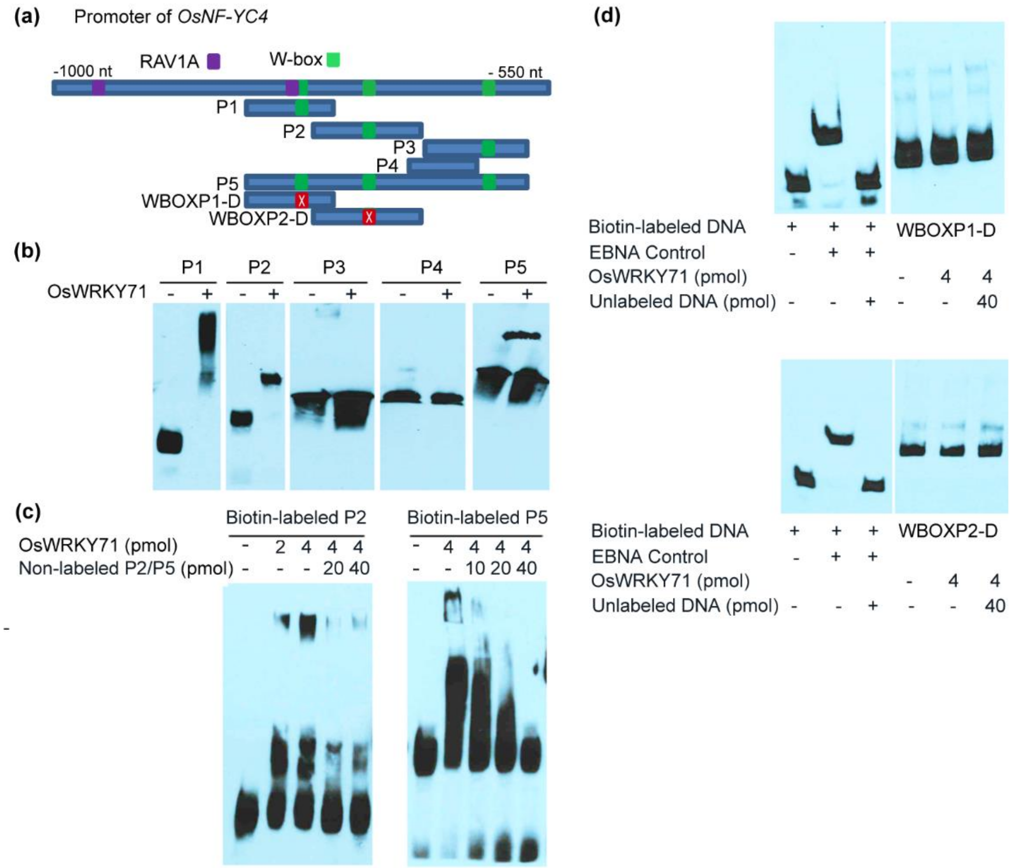

Fig. S5 The WRKY family member OsWRKY71 bound to the first and the second WRKY binding sites in the *OsNF-YC4* promoter. (a) Structure of *OsNF-YC4* promoter fragments, designed with different combinations for each W-box. These promoter fragments were PCR-amplified from the WT sibling plants and used for biotin labeling and EMSA (Electrophoretic Mobility Shift Assay) in b–c. (b) EMSA demonstrated the ability of OsWRKY71 to bind to both the 1st (in P1) and the 2nd W-box (in P2) in the *OsNF-YC4* promoter, while no binding was observed to the 3rd W-box (in P3). P4 served as a negative control lacking any W-box, while P5 contained all three W-box motifs. In b, 4 pmol OsWRKY71 was used in this experiment. (c) Competition experiments with biotin-unlabeled fragments confirmed the binding of OsWRKY71 to the 2nd W-box (P2 and P5) motif in the *OsNF-YC4* promoter. Three technical replicates were conducted for each experiment, using 20 fmol of biotin-labeled DNA fragments. (d) OsWRKY71 bound to both the 1st (in P1) and the 2nd W-box (in P2) in the *OsNF-YC4* promoter, as evidenced by EMSA. No binding was observed with the WBOXP1-D synthesized fragment lacking the 1st W-box or the WBOXP2-D synthesized fragment lacking the 2nd W-box (shown as red boxes in a). Twenty fmol of biotin-labeled DNA fragments were used in d. EBNA (Epstein-Barr nuclear antigen) from the EMSA kit was used as the positive control.

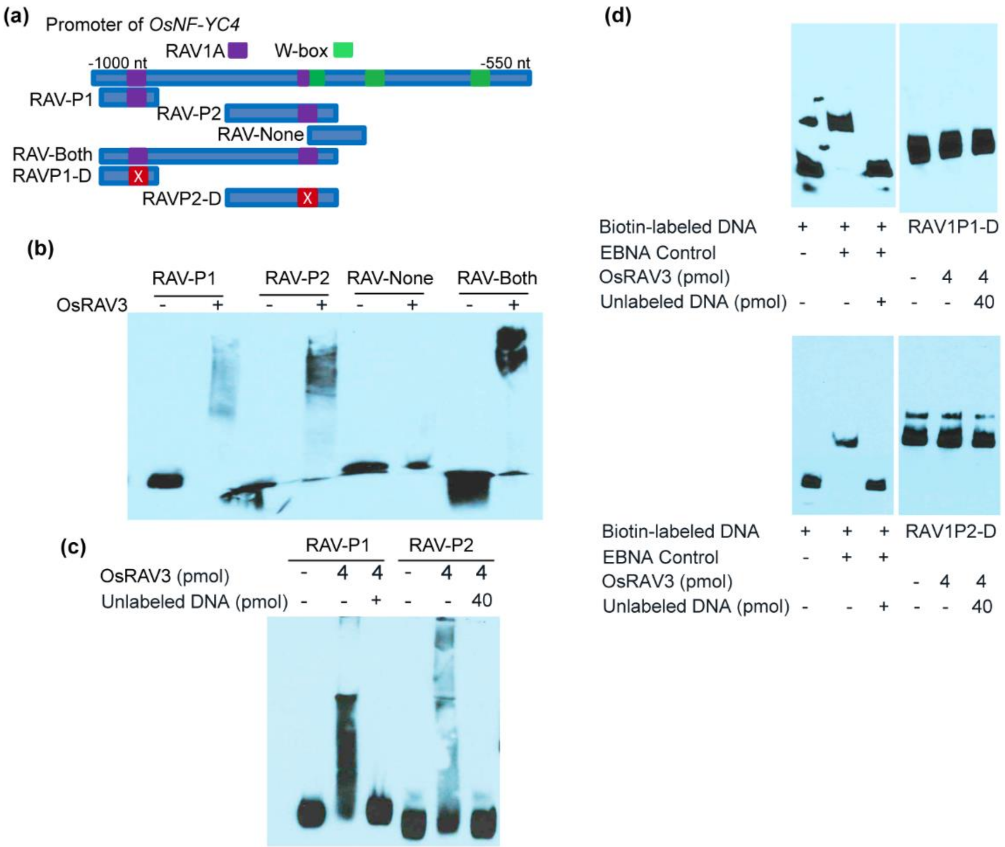

Fig. S6 The RAV family member OsRAV3 binds to the first and second RAV1A binding motifs (TGTTG) in the *OsNF-YC4* promoter. (a) Structures of *OsNF-YC4* promoter fragments, designed with different combinations for each RAV1A motif. These promoter fragments were obtained from CRISPR sibling plants by PCR and used for biotin labeling and EMSA (Electrophoretic Mobility Shift Assay) in b–c. RAV-None, lacking any RAV1A motif, served as a negative control. (b) EMSA demonstrated the binding affinity of OsRAV3 to both RAV1A motifs in the promoter of *OsNF-YC4*, whereas no binding was observed to the RAV-None fragment lacking any RAV1A motif. (c) Competition experiment with biotin-unlabeled fragments confirmed binding of OsRAV3 to both RAV1A motifs in the *OsNF-YC4* promoter. Biotin-labeled DNA fragments (20 fmol) were used in the experiment, along with 4 pmol of OsRAV3 in both b and c, and 4 pmol of unlabeled DNA fragments in c. (d) OsRAV3 did not bind to the RAVP1-D synthesized fragment lacking the 1st RAV1A motif and the RAVP2-D synthesized fragment lacking the 2nd RAV1A motif (shown as red boxes in a). Twenty fmol of biotin-labeled DNA fragments were used in d. EBNA (Epstein-Barr nuclear antigen) from the EMSA kit was used as the positive control in d.

GmNF-YC4-1 promoter:

ACACAAAAGTGACAAAATGTGGACTTGAAAATCACATGCTAGAGCAAAGCAAGGTTCAGATGA
AGCCAAATGGCTAGAACGTAAATGCTGCAAAAATCAATTATATTTGAATCTCACGGCATTGA
ACAAAAGAGAATGGACCCATCAATGGGGCATTGACATGGATGGATGGAACACCCTCAT**AGTCA**
CATGCCA**CAACA**GAGTAACGCTAAACAAAATGTATTAACAAAAACTATAGGATCATGCTTTCT
TAATTTAAAAACATAGGT**GGTCA**GTTTTTGTTAACATTAATTTTTAGGATTATT**TGTTG**AAGA
CAAATATGGAAGGTTTATAATAATTTATGTGCATTACTCAATGTAATCCTTAAAATGAAAATA
ACTAAACTTTTTTTTTTTGGATAGTTAAACAACTACGAAGCTAAATGAATAATTAAATCAATA
TTAATTTTGATTAAAAATAAGATTAATAATATCCCTAATATTGTGAACAAACTT**TGTTG**GTAA
TGTAAAAAAAATTAAAAGAAACAAGATTAAATTACGTATTTAATAATTTAAGATTAA**TGTTG**T
TTAATTTGATTTTTTAAATATTTATCTTTCTTTTGAATTTGATTCTTTAGTTCACTTAAGATT
ATGATCGTTAATAATTCTAAAAGATAAATGTCATCAATCTTGAATTGATTGAAAGATTAAATT
AAATAAAAAGAATTAAGATAAATGATGAAAATACATTTAAAAAATAAGATGAAACAAAATGTG
TCATTTAACCAAAAAAAAAAAGGAAAAATAAATTAAAAAAAGGAACTTACCCTTCCCCTCAAAA
AAGAAAAGAAAAAAAAAAAAAGGAACTTACCCTTGGTTGTTCCGTTGAAATTGAAAAAACAAACC
CTAAACTTACCTTAACCTAGGTCCTAGGGCACTGGTCGGATATGTTATTGTTTAGTTTACCTT
ATCCCACCACATACATAGTTTTTTTTTTCTTAAATTTCCCAATCAATTCCATCCATCGGGTCT
TTACTCTCTTACCCAACCCACACACACTCTTTCTCTCTCTCTCTTTCCCTGATCATCAAAATC
AGAAAAAATTGGGGGA

GmNF-YC4-1 promoter-P1：

GGCATTGACATGGATGGATGGAACACCCTCAT**AGTCA**CATGCCA**CAACA**GAGTAACGCTAAAC
AAAATGTATTAACAAAAACTATAGGATCATGCTTTCTTAATTTAAAAACATAGG

GmNF-YC4-1 promoter-P2：

CATGCTTTCTTAATTTAAAAACATAGGT**GGTCA**GTTTTTGTTAACATTAATTTTTAGGATTAT
T**TGTTG**AAGACAAATATGGAAGGTTTATAATAATTTATGTGCATTACTCAATGTAATCC

GmNF-YC4-1 promoter-P3:

GATTAATAATATCCCTAATATTGTGAACAAACTT**TGTTG**GTAATGTAAAAAAAATTAAAAGAA
ACAAGATTAAATTACGTATTTAATAATTTAAGATTAA

GmNF-YC4-1 promoter-P4:

TAAAAGAAACAAGATTAAATTACGTATTTAATAATTTAAGATTAA**TGTTG**TTTAATTTGATTT
TTTAAATATTTATCTTTCTTTTGAATTTGATTCTTTAGTTCACTTAAG

GmNF-YC4-1 promoter-P5:

GGCATTGACATGGATGGATGGAACACCCTCAT**AGTCA**CATGCCA**CAACA**GAGTAACGCTAA
ACAAAATGTATTAACAAAAACTATAGGATCATGCTTTCTTAATTTAAAAACATAGGT**GGT
CA**GTTTTTGTTAACATTAATTTTTAGGATTATT**TGTTG**AAGACAAATATGGAAGGTTTATA
ATAATTTATGTGCATTACTCAATGTAATCC

GmNF-YC4-1 promoter-P6:

CCCTAATATTGTGAACAAACTT**TGTTG**GTAATGTAAAAAAAATTAAAAGAAACAAGATTAAAT
TACGTATTTAATAATTTAAGATTAA**TGTTG**TTTAATTTGATTTTTTAAATATTTATCTTTCTT
TTGAATTTGATTCTTTAGTTCAC

GmNF-YC4-1 promoter-P7:

CATGCTAGAGCAAAGCAAGGTTCAGATGAAGCCAAATGGCTAGAACGTAAATGCTGCAAAAAT
CAATTATATTTGAATCTCACGGCATTGAACAAAAGAGAATGGACCCATCAATGGGG

GmNF-YC4-1 promoter-200-bp:

TTGACATGGATGGATGGAACACCCTCAT~~**AGTCA**~~CATGCCA**CAACA**GAGTAACGCTAAACAAAA
TGTATTAACAAAAACTATAGGATCATGCTTTCTTAATTTAAAAACATAGGT**GGTCA**GTTTTTG
TTAACATTAATTTTTAGGATTATT**TGTTG**AAGACAAATATGGAAGGTTTATAATAATTTATGT
GCATTACTCAATGTAA

Fig. S7 Sequence information of fragments from the promoter of *GmNF-YC4-1* used for EMSAs (Electrophoretic Mobility Shift Assay). RAV1A target motif is shown in purple font, while the WRKY target motif (W-box) is shown in green font. Struck-through letters indicate 5-bp deletions.

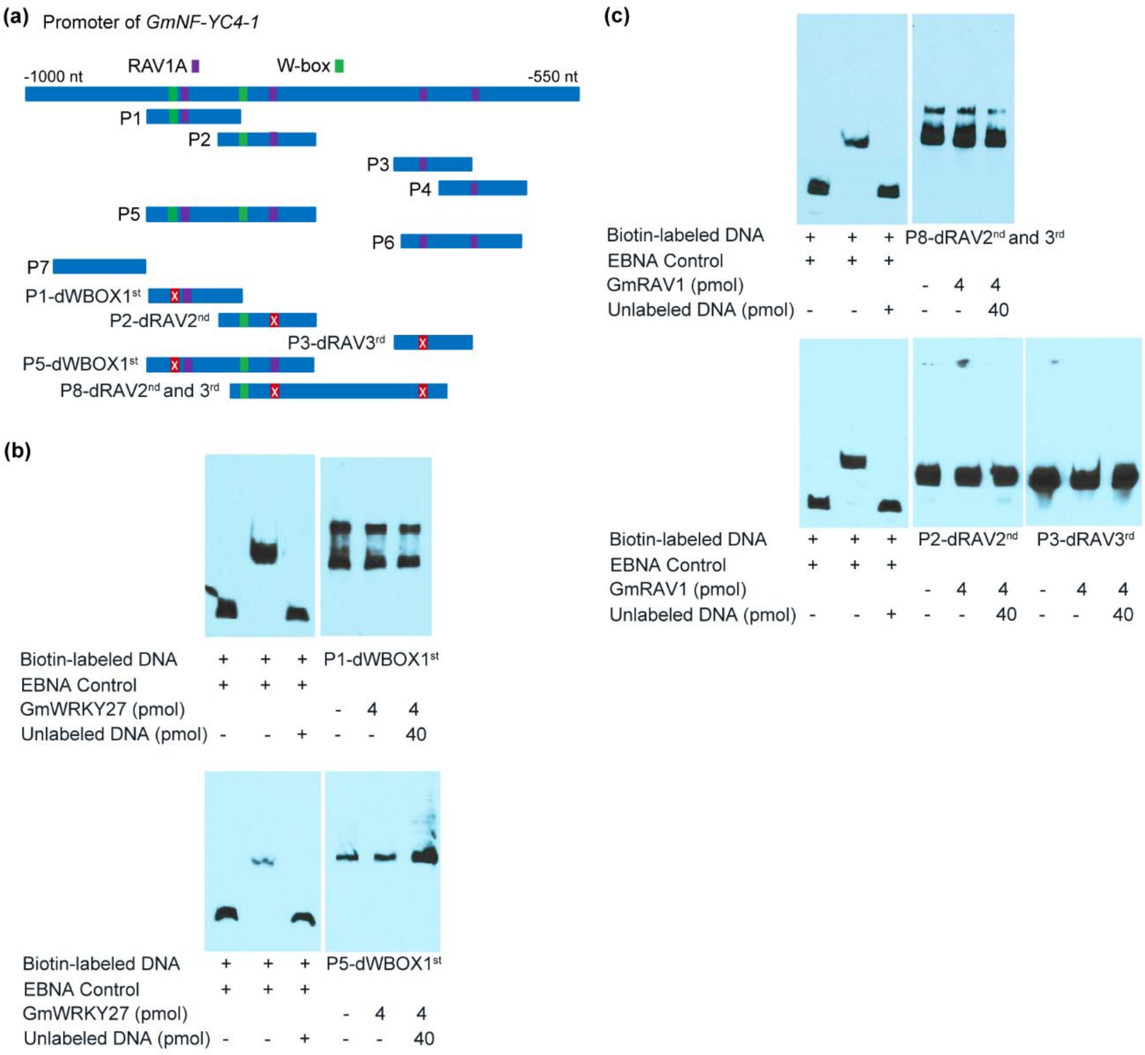

Fig. S8 GmRAV1 and GmWRKY27 bind to RAV1A and WRKY motifs in the promoter of *GmNF-YC4-1*. (a) Structures of *GmNF-YC4-1* promoter fragments, designed with different combinations of each RAV and W-box motif. These promoter fragments were PCR-amplified from WT plants and used for biotin labeling and EMSA (Electrophoretic Mobility Shift Assay) in b and c. (b) GmWRKY27 was unable to bind to P1-dWBOX1st, and P5-dWBOX1st, which lacked the 1st WBOX (5-bp; shown as red box in a). (c) GmRAV1 could not bind to either the 2nd (in P2) or the 3rd RAV1A motif (in P3) in the promoter of *GmNF-YC4-1*. Similarly, it did not bind to the P2-dRAV2nd or P3-dRAV3rd synthesized fragments lacking the 2nd or the 3rd RAV1A motif, respectively. Additionally, P8-dRAV2nd and 3rd, lacking both the 2nd and 3rd RAV1A motifs (5-bp; shown as red boxes in a), also showed no binding. Twenty fmol of biotin-labeled DNA fragments were used in b and c. EBNA (Epstein-Barr nuclear antigen) from the EMSA kit was used as the positive control in b and c.

p*OsNF-YC4*:

PCR Primer-F

TGCATGGATGCGTGAATCTAT**TGTTG**CATTTTTTCTAATGGATAGTTAGATTTGTTCCAGC
TTTTTTTTTGTCAATTCTGTTACATTTGCAGTGTCTTCAACTTCTGCATCATTCTTCTCT
ATTCTTCCTTCACTTTCTTTCTTGAACTAGAAAAGCAAACCAGAGTTCTTTTTCCCCC
TCTTCTACATA^**AACGAAAACAGCT**TGTTGACTGGCTCCCTAGAGCTTTTTGTAAGT
TGATCATCGAAGTAGCTAGTTCTCTTCACTTATC**AGTCA**TCACTGTTCTATGTTCTATCT
GCATTTTCCTTGATTTTGTACTTTTCCTGAACGAAAGGACAATCCTTAGCCATCATAAT
GCTATGATC**GACTTATTCTGAAGTC^ATC**CGGCCTCGATCCTCTTTTGTTTCGGTGAG
ATCTGTAATGGTTTAGGAAAATTATGGATCTCTGAAAAATAAGAAACATCTAGTAGTA
TATGAAATTAAGAAATGTCGGACCACGTCAAATCCTACTGGTATCATATACCAGATAA
CTAACTTTTTGAATGGAACCAAACACGAGATTGTGAATATATAGCAGTAAGAAATAC
AACCCCGAAGGGT

PCR Primer-R

Purple: RAV1A

Green: W-box

^: CRISPR cut site

Fig. S9 Guide RNA design for the *OsNF-YC4* promoter. Two guide RNAs were designed and constructed to edit the *OsNF-YC4* promoter. The sequences are underlined and accompanied with 5’-NGG (highlighted in red font), as protospacer-adjacent motifs (PAMs) for Cas9/gRNA cleavage sites in the *OsNF-YC4* promoter. The PCR primers (highlighted in blue font) were used to assay plants for gene-edits.

New Phytologist

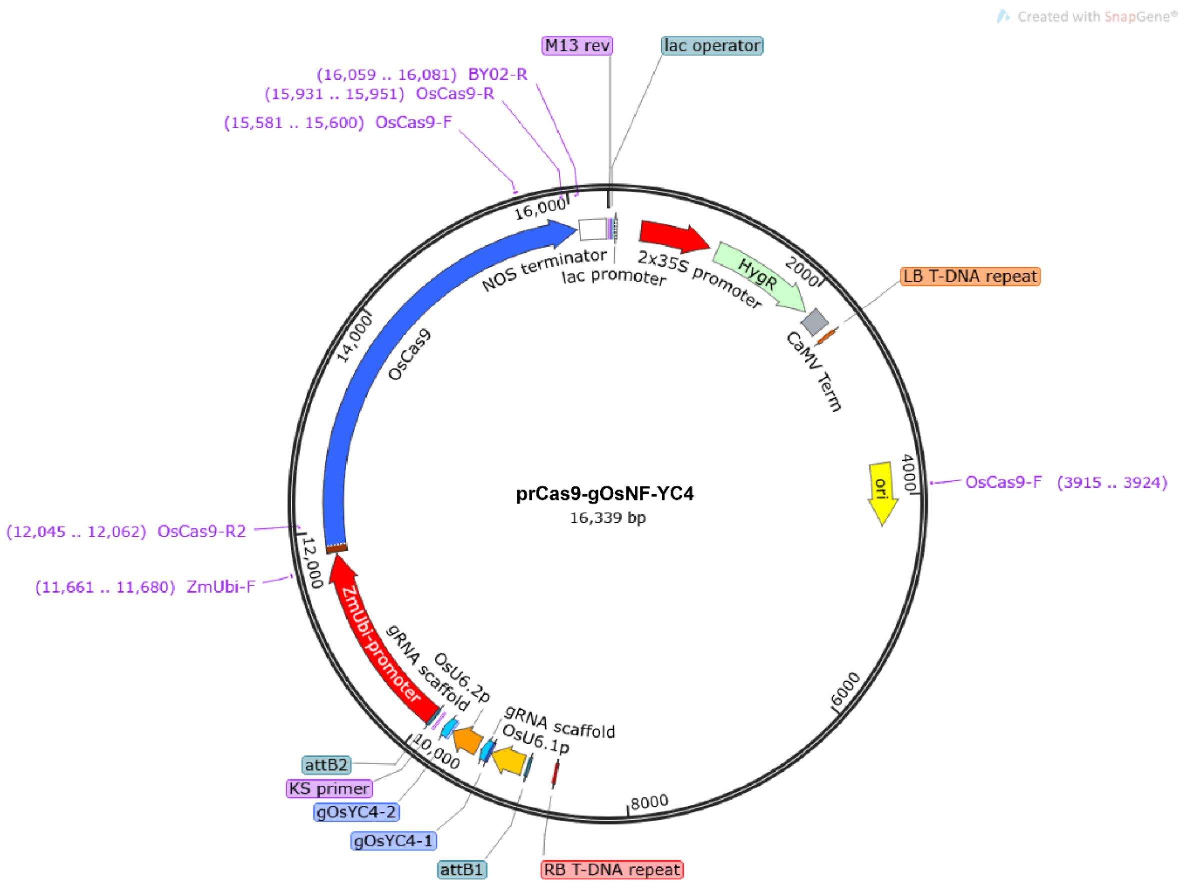

Fig. S10 Map of the CRISPR/Cas9 construct prCas9-gOsNF-YC4 for editing the *OsNF-YC4* gene promoter in rice.

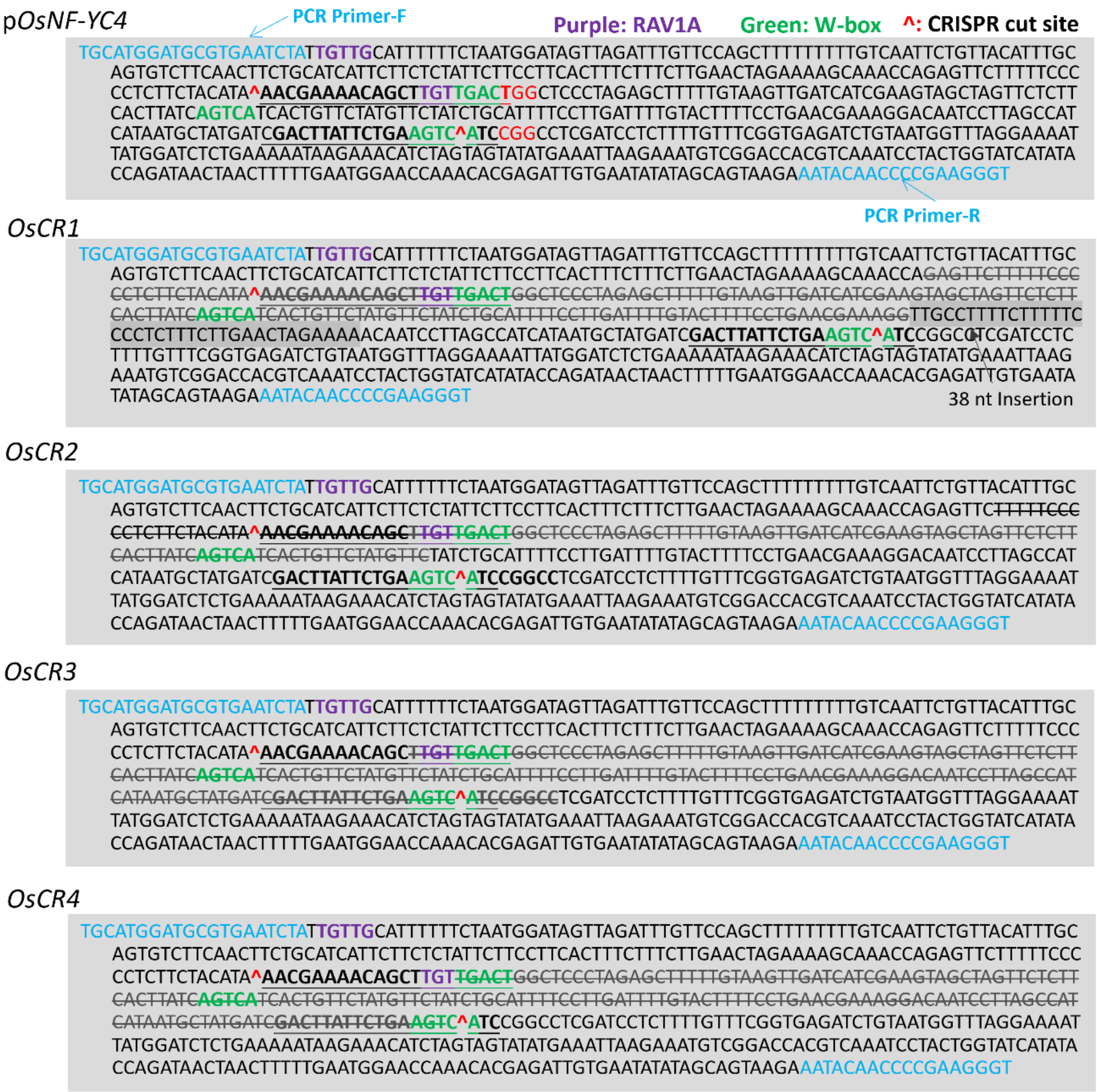

Fig. S11 Sequences of the *OsNF-YC4* promoters in CRISPR-edited rice plants. Two sequences in bold and underlined are designed for Cas9/gRNA cleavage sites. The primers (blue font) were used to check mutations/deletions. "~~XXX~~" indicates the deletions in the promoter of *OsNF-YC4* in CRISPR-edited rice plants. Four types of sequences (OsCR1-OsCR4) were generated within the edited materials.

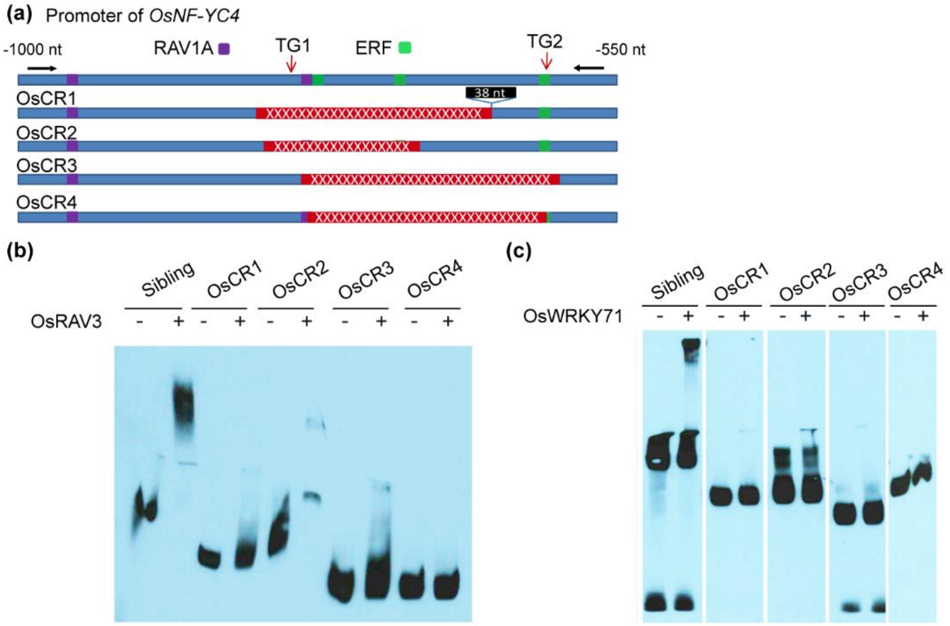

Fig. S12 OsWRKY71/OsRAV3 lost or significantly reduced binding to the promoter of *OsNF-YC4* from CRISPR/Cas9-edited plants when the first and second W-box motifs and the second RAV1A motif were deleted. (a) Structures of promoters in four gene-edited events with different deletions. Red rectangle indicates homozygous deletion. Arrows indicate the location of the primers used for PCR amplification and biotin labeling for EMSA (Electrophoretic Mobility Shift Assay) experiments in b and c. (b) EMSA demonstrated that OsRAV3 lost or had significantly reduced binding affinity to the *OsNF-YC4* promoter when the 2nd RAV1A motif was deleted. (c) OsWRKY71 lost/significantly reduced binding affinity to the *OsNF-YC4* promoter when the 1st and 2nd W-box motifs were deleted. Three technical replicates were conducted for each experiment. Biotin-labeled DNA fragments (20 fmol) were used in the experiments. In b and c, 4 pmol of OsRAV3 or OsWRKY71 was added for the experiments.

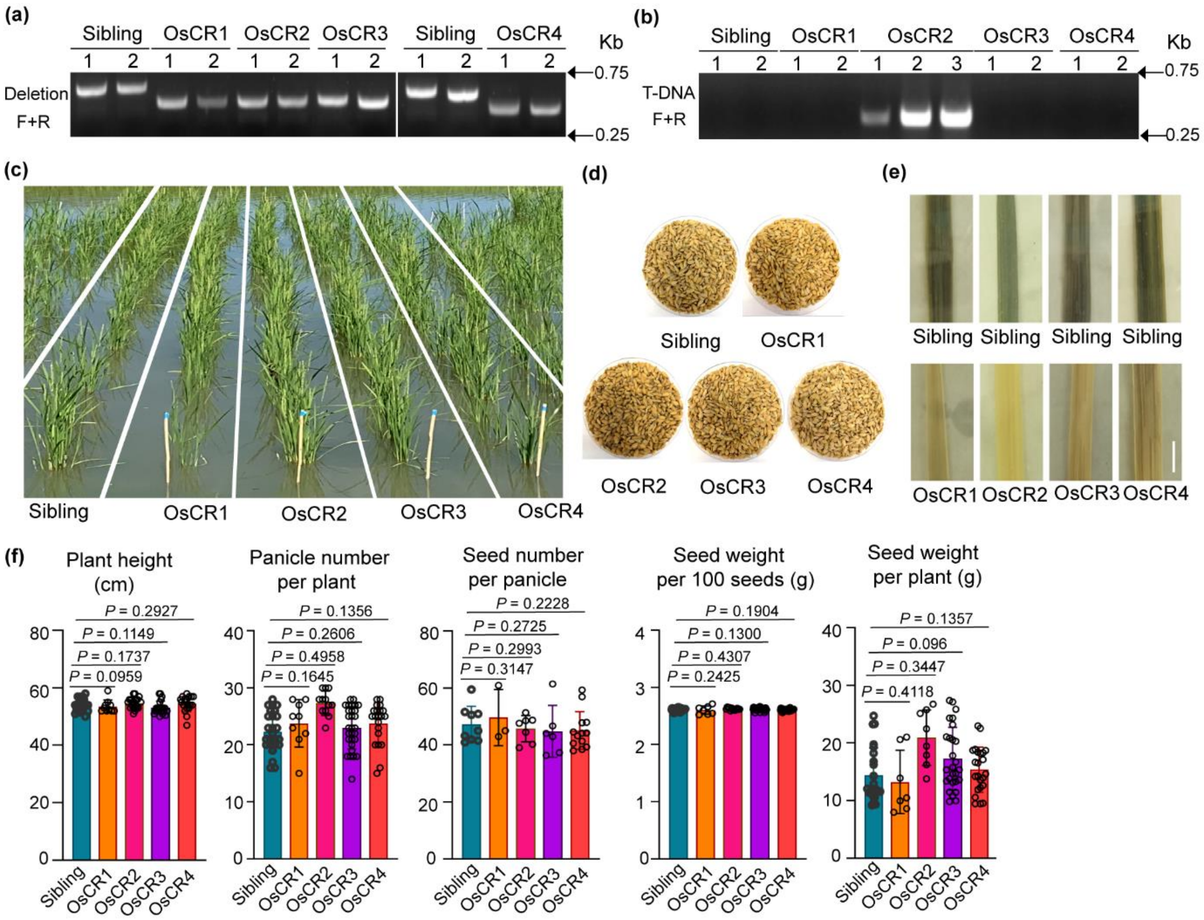

Fig. S13 Leaf starch content was decreased in otherwise morphologically similar CRISPR/Cas9-edited rice plants with deletions in RAV1A/ W-box in the *OsNF-YC4* promoter. (a, b) Genotyping of T2 plants using PCR to detect *OsNF-YC4* promoter deletions and the presence or absence of the T-DNA vector in assayed plants. (c, d) Phenotype of CRISPR/Cas9-edited rice plants and seeds. (e) Leaf starch content observed through starch staining. Starch staining was conducted on five plants per line. Scale bar, 1 cm. (f) Evaluation of plant height, panicle number per plant, seed number per panicle, seed weight per 100 seeds or per plant to assess the effect of CRISPR/Cas9-edited rice plants compared to sibling controls. Data were recorded for the T2 generation of field-grown, CRISPR-edited mutant plants and the T3 generation of field-grown CRISPR-edited mutant seeds. All data in bar charts show mean values ± error bars indicating the standard error of mean for three biological replicates. Student's *t*-test was used to compare CRISPR/Cas9-edited rice plants to controls; $n \geq 3$ plants; $P > 0.05$ for these tests.

PCR primer-F

p*GmNF-YC4-1*:

GTGACAAAATGTGGACTTGAAAATCACATGCTAGAGCAAAGCAAGGTTCAGATGAAGCCAA
ATGGCTAGAACGTAAATGCTGCAAAAATCAATTATATTTGAATCTCACGGGCATTGAACAAAA
GAGAATGGACCCATCAATGGGGCATTGACATGGATGGATGGAACACCCTCAT^AGTCACAT
GCCACAA^CAGAGTAACGCTAAACAAAATGTATTAACAAAAACTATAGGATCATGCTTTCTTA
ATTTAAAAA^CATAGGTGGTCAGTTTTTGTTAACATTAATT^TTTAGGATTATTTGTTGAAGAC
AA^ATATGGAAGGTTTATAATAATTTATGTGCATTACTCAATGTAATCCTTAAAATGAAAATAAC
TAAACTTTTTTTTTTTGGATAGTTAAACAACTACGAAGCTAAATGAATAATTAAATCAATATTA
ATTTTGATTAAAAATAAGATTAATAATATCCCTAATATTGTGAACAAACTT^TGTTGGTAATGTA
AAAAAAATTAAAAGAAACAAGATTAAATTACGTATTTAATAATTTAAGATTAATGTTGTTTAAT
TTGATTTTTTAAATATTTATCTTTCTTTTGAATTTGATTCTTTAGTTCACTTAAGATTATGATCGT
TAATAATTCTAAAAGATAAATGTCATCAATCTTGAATTGATTGAAAGATTAAATTAAATAAAAA
GAATTAAGATAAATGATGAAAATACATTTAAAAAATAAGATGAAACAAAATGTGTCATTTAAC
CAAAAAAAAAAGGAAAAATAAATTAAAAAAAAGGAACTTACCCTTCCCCTCAAAAAAGAAAA
GAAAAAAAAAAAGGAACTTACCCTTGGTTGTTCCGTTGAAATTGAAAAAACAAACCCTAAA
CTTACCTTAACCTAGGTCCTAGGGCACTGGTCGGATATGTTATTGTTTAGTTTACCTTATCCCA
CCACATACATAGTTTTTTTTTTCTTAAATTTCCCAATCAATTCCATCCATCGGGTCTTTACTCTC
TTACCCAACCCACACACACTCTTTCTCTCTCTCTCTTTCCCTGATC

PCR primer-R

Purple: RAV1A

Green: W-box

^: CRISPR cut site

Fig. S14 Guide RNAs designed for the *GmNF-YC4-1* promoter. Six guide RNAs were designed and constructed to edit the *GmNF-YC4-1* promoter. The sequences of the guide RNAs are underlined, with Guide RNAs 1 and 2 overlapping, and are accompanied by 5’-NGG motifs (red font), designed for Cas9/gRNA cleavage sites in the *GmNF-YC4-1* promoter. The primers (blue font) were used to check mutations/deletions.

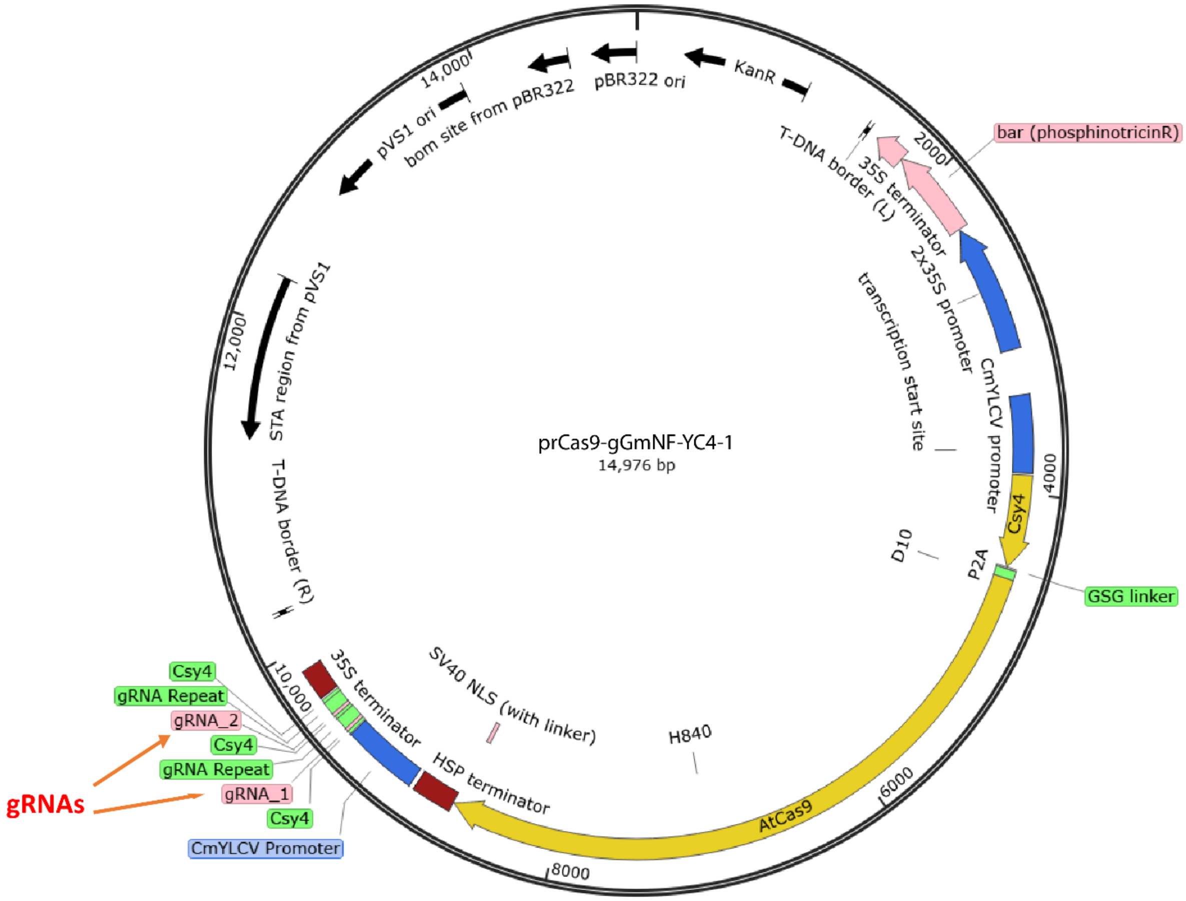

Fig. S15 Map of the CRISPR/Cas9 construct prCas9-gGmNF-YC4-1 for editing the *GmNF-YC4-1* promoter in soybean.

p*GmNF-YC4-1*

PCR Primer-F

Purple: RAV1A Green: W-box ^: CRISPR cut site

```
GTGACAAAATGTGGACTTGAAAATCACATGCTAGAGCAAAGCAAGGTTCAGATGAAGCCAAATGGCTAGAACGTAAATGCTGCAAAAATCA
ATTATATTTGAATCTCACGGGCATTGAACAAAAGAGAATGGACCCATCAATGGGGCATTGACATGGATGGATGGAACACCCTCAT^AGTCAC
ATGCCACAA^CAGAGTAACGCTAAACAAAATGTATTAACAAAAACTATAGGATCATGCTTTCTTAATTTAAAAA^CATAGGTGGTCAGTTTT
TGTTAACATTAATT^TTTAGGATTATTTGTTGAAGACAA^ATATGGAAGGTTTATAATAATTTATGTGCATTACTCAATGTAATCCTTAAAATG
AAAATAACTAAACTTTTTTTTTTTGGATAGTTAAACAACTACGAAGCTAAATGAATAATTAAATCAATATTAATTTTGATTAAAAATAAGATTA
ATAATATCCCTAATATTGTGAACAAACTT^TGTTGGTAATGTAAAAAAAATTAAAAGAAACAAGATTAAATTACGTATTTAATAATTTAAGAT
TAATGTTGTTTAATTTGATTTTTTAAATATTTATCTTTCTTTTGAATTTGATTCTTTAGTTCACTTAAGATTATGATCGTTAATAATTCTAAAAGAT
AAATGTCATCAATCTTGAATTGATTGAAAGATTAAATTAAATAAAAAGAATTAAGATAAATGATGAAAATACATTTAAAAAATAAGATGAAA
CAAAATGTGTCATTTAACCAAAAAAAAAAAGGAAAAATAAATTAAAAAAAGGAACTTACCCTTCCCCTCAAAAAAGAAAAGAAAAAAAAAAA
GGAACTTACCCTTGGTTGTTCCGTTGAAATTGAAAAAACAAACCCTAAACTTACCTTAACCTAGGTCCTAGGGCACTGGTCGGATATGTTATT
GTTTAGTTTACCTTATCCCACCACATACATAGTTTTTTTTTTCTTAAATTTCCCAATCAATTCCATCCATCGGGTCTTTACTCTCTTACCCAACCCA
CACACACTCTTTCTCTCTCTCTCTTTCCCTGATC
```

PCR Primer-R

GmCR1

GTGACAAAATGTGGACTTGAAAATCACATGCTAGAGCAAAGCAAGGTTCAGATGAAGCCAAATGGCTAGAACGTAAATGCTGCAAAAATCA
ATTATATTTGAATCTCACGGGCATTGAACAAAAGAGAATGGACCCATCAATGGGGCATTGACATGGATGGATGGAACACCCTCAT^AGTCAC
ATGCCACAA^CAGAGTAACGCTAAACAAAATGTATTAACAAAAACTATAGGATCATGCTTTCTTAATTTAAAAA^~~CATAGGTGGTCAGTTTT~~
~~TGTTAACATTAATT^TTTAGGATTATTTGTTGAAGACAA~~^ATATGGAAGGTTTATAATAATTTATGTGCATTACTCAATGTAATCCTTAAAATG
AAAATAACTAAACTTTTTTTTTTTGGATAGTTAAACAACTACGAAGCTAAATGAATAATTAAATCAATATTAATTTTGATTAAAAATAAGATTA
ATAATATCCCTAATATTGTGAACAAACTT^TGTTGGTAATGTAAAAAAAATTAAAAGAAACAAGATTAAATTACGTATTTAATAATTTAAGAT
TAATGTTGTTTAATTTGATTTTTTAAATATTTATCTTTCTTTTGAATTTGATTCTTTAGTTCACTTAAGATTATGATCGTTAATAATTCTAAAAGAT
AAATGTCATCAATCTTGAATTGATTGAAAGATTAAATTAAATAAAAAGAATTAAGATAAATGATGAAAATACATTTAAAAAATAAGATGAAA
CAAAATGTGTCATTTAACCAAAAAAAAAAAGGAAAAATAAATTAAAAAAAGGAACTTACCCTTCCCCTCAAAAAAGAAAAGAAAAAAAAAAA
GGAACTTACCCTTGGTTGTTCCGTTGAAATTGAAAAAACAAACCCTAAACTTACCTTAACCTAGGTCCTAGGGCACTGGTCGGATATGTTATT
GTTTAGTTTACCTTATCCCACCACATACATAGTTTTTTTTTTCTTAAATTTCCCAATCAATTCCATCCATCGGGTCTTTACTCTCTTACCCAACCCA
CACACACTCTTTCTCTCTCTCTCTTTCCCTGATC

Fig. S16 Sequences in the *GmNF-YC4-1* promoters in soybean CRISPR-edited plants. Six sequences in bold and underlined were designed for Cas9/gRNA cleavage sites. The primers (blue font) were used to check mutations/deletions. The RAV1A target motif is in purple font, while the WRKY target motif is in green font. "~~XXX~~" indicates the deletions in the promoter of *GmNF-YC4-1* in CRISPR-edited plants.

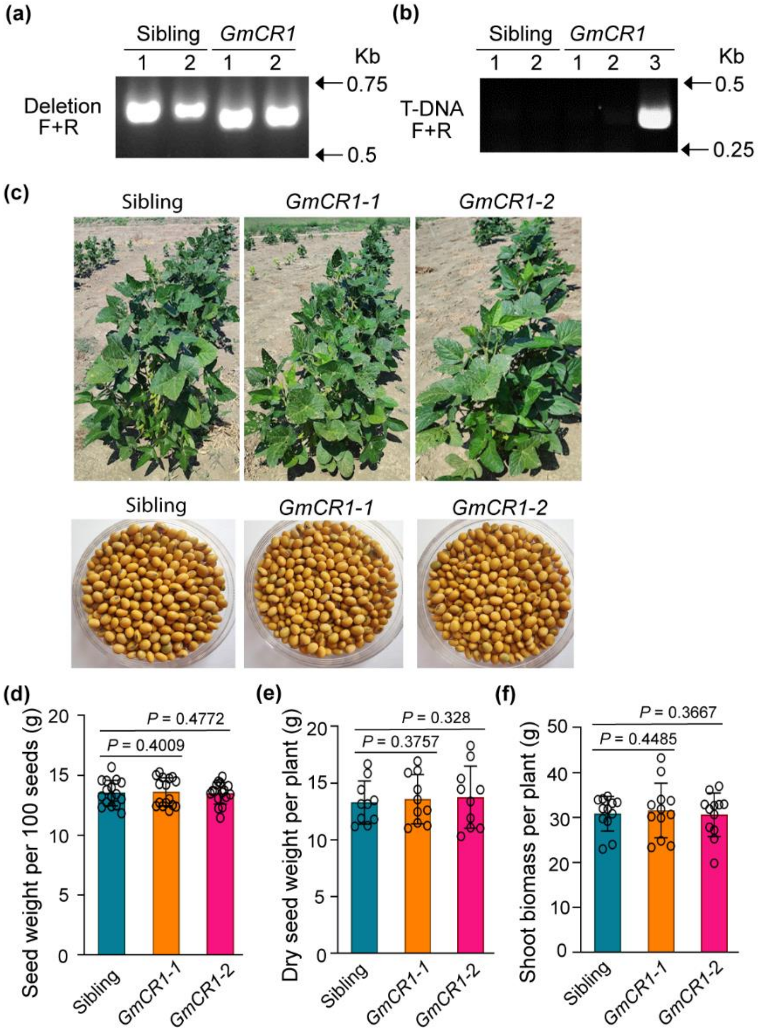

Fig. S17 Morphological assessment of CRISPR/Cas9-edited soybean plants with deletions in RAV1A/ W-box in the *GmNF-YC4-1* promoter, compared to WT siblings. (a, b) Genotyping of T2 plants using PCR to detect GmCR mutant lines. Two T-DNA free lines, GmCR1-1, GmCR1-2, were selected, while T-DNA line GmCR1-3 was used as a control. (c) Phenotype of CRISPR/Cas9-edited soybean plants and seeds. (d) Evaluation of seed weight per 100 seeds. (e) Evaluation of dry seed weight per plant. (f) Assessment of mature shoot biomass to evaluate the effect of CRISPR/Cas9-edited soybean plants compared to sibling controls. Data were recorded for the T3 generation of field-grown CRISPR-edited seeds. All data in bar charts show mean values ± error bars indicating the standard error of mean. Student's *t*-test was used to compare CRISPR/Cas9-edited soybean plants to controls; $n \geq 10$ plants; $P > 0.05$ for these tests.

(a) Expression pattern of *AtNF-YC4*

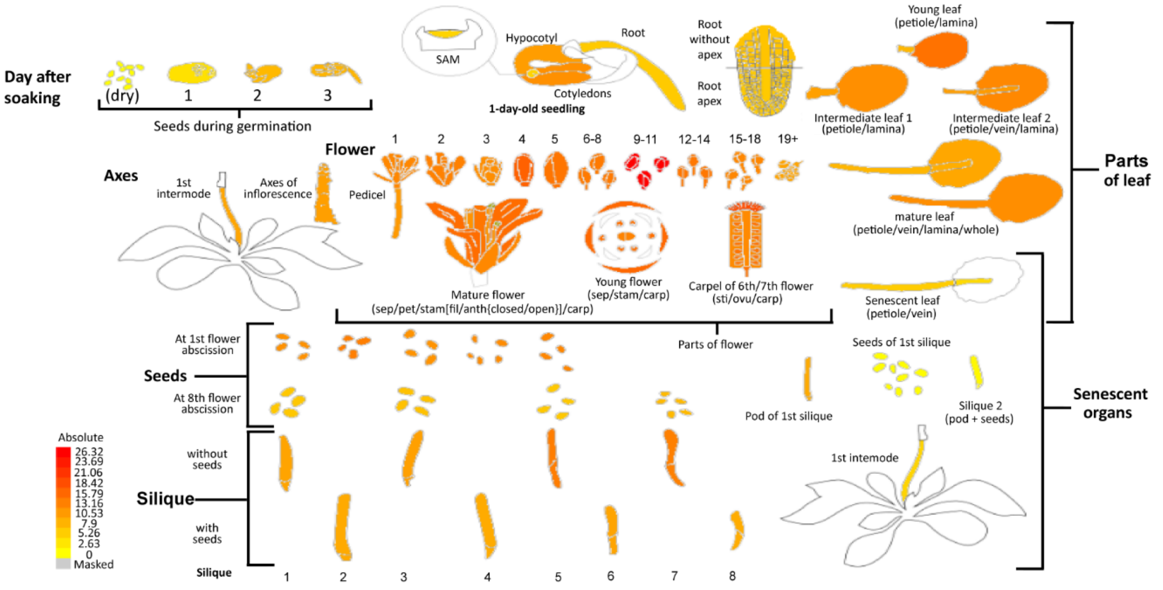

Data from A high resolution map of the Arabidopsis thaliana developmental transcripttome based on RNA-Seq profiling by Klepikova et al., 2016.

(b) Expression pattern of *OsNF-YC4*

RNA-Seq FPKM Expression Values

Library Name	Library Description	Rice Genotype	FPKM
OSN_AA	Leaves-20 days	Nipponbare	145.946
OSN_AB	Post-emergence inflorescence	Nipponbare	35.365
OSN_AC	Pre-emergence inflorescence	Nipponbare	5.23059
OSN_AD	Anther	Nipponbare	0
OSN_AE	Pistil	Nipponbare	12.3476
OSN_AF	Seed-5 DAP	Nipponbare	8.42952
OSN_AG	Embryo- 25 DAP	Nipponbare	0
OSN_AH	Endosperm- 25 DAP	Nipponbare	0
OSN_AK	Seed- 10 DAP	Nipponbare	0
OSN_BH	Endosperm- 25 DAP (replicate)	Nipponbare	0
OSN_CA	Leaves- 20 days (replicate)	Nipponbare	184.189
SRR042529	Shoots	Nipponbare	34.352

TRAP-Seq FPKM Expression Values

	RNA-Seq FPKM	TRAP-Seq FPKM
Seedling	45.7849	22.1974
Callus	0.033156	0.0664363
Panicles	0.480718	0.515118

(c) Expression pattern of *GmNF-YC4-1*

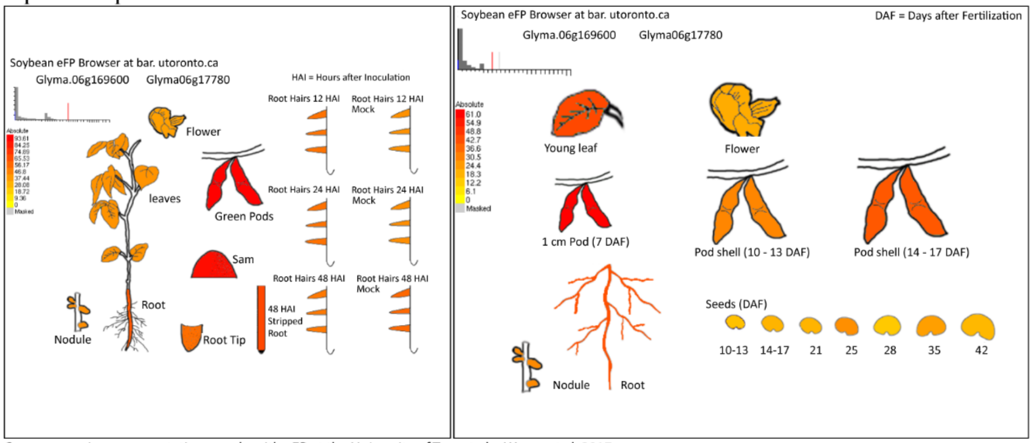

Gene expression representations made with eFP at the University of Toronto by Waese et al. 2017.

Fig. S18 Expression pattern of *AtNF-YC4*, *OsNF-YC4* and *GmNF-YC4-1* from online public databases. (a) Expression pattern of *AtNF-YC4* from the TAIR website (http://bar.utoronto.ca/efp/cgi-bin/efpWeb.cgi?primaryGene=AT5G63470&dataSource=Klepikova_Atlas&modeInput=Absolute). (b) Expression pattern of *OsNF-YC4* from the Rice Genome Annotation Project Team website (http://rice.uga.edu/cgi-bin/ORF_infopage.cgi?orf=LOC_Os03g14669). (c) Expression pattern of *GmNF-YC4-1* from the SoyBase website (https://www.soybase.org/expression/expression_explorer2.php?glyma=Glyma06g17780).

New Phytologist

Table S1 List of primers and their applications.

Primer name	Sequence (5' to 3')	Purpose
OsTUB-qPCR-F	GCTTGCTACCCCTACCTTTG	Control gene for transcript level
OsTUB-qPCR-R	GACCAGGGAACCTCAGACAG	Control gene for transcript level
OsNF-YC4-qPCR-F	CAACCAGCAGCTACCCTACG	*OsNF-YC4* transcript level
OsNF-YC4-qPCR-R	CTGCTGGAGCAGGTGGTG	*OsNF-YC4* transcript level
D1-pOsNF-YC4-LUC-F1	CCCGGATCCGCAAGAACCCCAGTTGAG AGTGG	D1 type of deletion for pOsNF-YC4-LUC
D1-pOsNF-YC4-LUC-R1	CTAACTATCCATTAGAAAAAATGATAGA TTCACGCATCCATGCATGT	D1 type of deletion for pOsNF-YC4-LUC
D1-pOsNF-YC4-LUC-F2	ACATGCATGGATGCGTGAATCTATCATT TTTTCTAATGGATAGTTAG	D1 type of deletion for pOsNF-YC4-LUC
D1-pOsNF-YC4-LUC-R2	CCCAAGCTTTGTCACCTATATATACGTA CCTTA	D1 type of deletion for pOsNF-YC4-LUC
D2-pOsNF-YC4-LUC-F1	CCCGGATCCGCAAGAACCCCAGTTGAG AGTGG	D2 type of deletion for pOsNF-YC4-LUC
D2-pOsNF-YC4-LUC-R1	CTTACAAAAAGCTCTAGGGAGCCAGCT GTTTTCGTTTATGTAGAAGA	D2 type of deletion for pOsNF-YC4-LUC
D2-pOsNF-YC4-LUC-F2	TCTTCTACATAAACGAAAACAGCTGGCT CCCTAGAGCTTTTTGTAAG	D2 type of deletion for pOsNF-YC4-LUC
D2-pOsNF-YC4-LUC-R2	CCCAAGCTTTGTCACCTATATATACGTA CCTTA	D2 type of deletion for pOsNF-YC4-LUC
D3-pOsNF-YC4-LUC-F1	CCCGGATCCGCAAGAACCCCAGTTGAG AGTGG	D3 type of deletion for pOsNF-YC4-LUC
D3-pOsNF-YC4-LUC-R1	AAACAAAAGAGGATCGAGGCCGGAGA TAAGTGAAGAGAACTAGCTACT	D3 type of deletion for pOsNF-YC4-LUC
D3-pOsNF-YC4-LUC-F2	AGTAGCTAGTTCTCTTCACTTATCTCCG GCCTCGATCCTCTTTTGTTT	D3 type of deletion for pOsNF-YC4-LUC
D3-pOsNF-YC4-LUC-R2	CCCAAGCTTTGTCACCTATATATACGTA CCTTA	D3 type of deletion for pOsNF-YC4-LUC
pOsNF-YC4-P1-F	ACCAGAGTTCTTTTTCCCCCTC	P1 at *OsNF-YC4* promoter Used for EMSA
pOsNF-YC4-P1-R	AGCTACTTCGATGATCAACTTACA	P1 at *OsNF-YC4* promoter Used for EMSA
pOsNF-YC4-P2-F	TGGCTCCCTAGAGCTTTTTGT	P2 at *OsNF-YC4* promoter Used for EMSA
pOsNF-YC4-P2-R	AAGGATTGTCCTTTCGTTCAGG	P2 at *OsNF-YC4* promoter Used for EMSA
pOsNF-YC4-P3-F	TCCTGAACGAAAGGACAATC	P3 at *OsNF-YC4* promoter Used for EMSA
pOsNF-YC4-P3-R	ACAAAAGAGGATCGAGGCCG	P3 at *OsNF-YC4* promoter Used for EMSA
pOsNF-YC4-P4-F	TCTGCATTTTCCTTGATTTTGTACT	P4 at *OsNF-YC4* promoter Used for EMSA
pOsNF-YC4-P4-R	AGCATTATGATGGCTAAGGATTGT	P4 at *OsNF-YC4* promoter Used for EMSA
pOsNF-YC4-P5-F	ACCAGAGTTCTTTTTCCCCCTC	P5 at *OsNF-YC4* promoter Used for EMSA

pOsNF-YC4-P5-R	ACAAAAGAGGATCGAGGCCG	P5 at *OsNF-YC4* promoter Used for EMSA
pOsNF-YC4-198bp-F	AACAGCTTGTTGACTGGCTCCCTA	PCR for 198bp fragment at *OsNF-YC4* promoter for EMSA
pOsNF-YC4-198bp-R	AGGATCGAGGCCGGATGACT	PCR for 198bp fragment at *OsNF-YC4* promoter for EMSA
OsWRKY121-F	GGGGACAAGTTTGTACAAAAAAGCAGGCTTCATGGAGGGGATGGAGGAAGC	OsWRKY121 expression in *E. coli*
OsWRKY121-R	GGGGACCACTTTGTACAAGAAAGCTGGGTCTCAGGCTGGCTGGGCAGCTA	OsWRKY121 expression in *E. coli*
OsRAV3-F	GGGGACAAGTTTGTACAAAAAAGCAGGCTTCATGGACAGCTCCAGCTGC	OsRAV3 expression in *E. coli*
OsRAV3-R	GGGGACCACTTTGTACAAGAAAGCTGGGTCCTATACTAGTGCCAGCTCTATGCA	OsRAV3 expression in *E. coli*
OsWRKY71-F-2	GGGGACAAGTTTGTACAAAAAAGCAGGCTTCATGGATCCGTGGATTAGCACCCAG	OsWRKY71 expression in *E. coli*
OsWRKY71-R	GGGGACCACTTTGTACAAGAAAGCTGGGTCTCAATCCTTGGTCGGCGAGAGC	OsWRKY71 expression in *E. coli*
OsCas9-F	GGGTAATGAACTCGCTCTGC	Genotype *OsCas9* gene, checking T-DNA insertion
OsCas9-R	TGGCGTCAAGAACTTCCTTTG	Genotype *OsCas9* gene, checking T-DNA insertion
OsNF-YC4-Del-F	TGCATGGATGCGTGAATCT	Genotype *OsNF-YC4* promoter mutations through PCR and sequencing
OsNF-YC4-Del-R	ACCCTTCGGGGTTGTATTTC	Genotype *OsNF-YC4* promoter mutations through PCR and sequencing
pOsNF-YC4-234F	TCTTGAACTAGAAAAGCAAAC	PCR for EMSA for Sibling and CRISPR-editing *OsNF-YC4* promoter mutants
pOsNF-YC4-510R	ACAGATCTCACCGAAACAAAA	PCR for EMSA for Sibling and CRISPR-editing *OsNF-YC4* promoter mutants
gRNA-OsNF-YC4-F1	TGTTGAACGAAAACAGCTTGTTGAC	gRNA targeting *OsNF-YC4* for promoter mutations
gRNA-OsNF-YC4-R1	AAACGTCAACAAGCTGTTTTCGTTC	gRNA targeting *OsNF-YC4* for promoter mutations
gRNA-OsNF-YC4-F2	GTGTGACTTATTCTGAAGTCATC	gRNA targeting *OsNF-YC4* for promoter mutations
gRNA-OsNF-YC4-R2	AAACGATGACTTCAGAATAAGTC	gRNA targeting *OsNF-YC4* for promoter mutations
GmRAV1F	GGGGACAAGTTTGTACAAAAAAGCAGGCTCCATGGATGCAATTAGTTGCCTGG	GmRAV1 expression in *E. coli*
GmRAV1R	GGGGACCACTTTGTACAAGAAAGCTGGGTCCTACAAAGCACCAATAATCTTAG	GmRAV1 expression in *E. coli*
GmWRKY27F	GGGGACAAGTTTGTACAAAAAAGCAGGCTTCATGCCAACGCTAACTATACCAA	GmWRKY27 expression in *E. coli*

GmWRKY27R	GGGGACCACTTTGTACAAGAAAGCTGG GTCCTAGTTGGTGGAGAAAGTAGTGC	GmWRKY27 expression in *E. coli*
pGmNF-YC4-1-P1-F	GGCATTGACATGGATGGATGG	P1 at *GmNF-YC4-1* promoter used for EMSA
pGmNF-YC4-1-P1-R	CCTATGTTTTTAAATTAAGAAAGCATG	P1 at *GmNF-YC4-1* promoter used for EMSA
pGmNF-YC4-1-P2-F	CATGCTTTCTTAATTTAAAAACATAGG	P2 at *GmNF-YC4-1* promoter used for EMSA
pGmNF-YC4-1-P2-R	GGATTACATTGAGTAATGCAC	P2 at *GmNF-YC4-1* promoter used for EMSA
pGmNF-YC4-1-P3-F	GATTAATAATATCCCTAATATTGTGAA	P3 at *GmNF-YC4-1* promoter used for EMSA
pGmNF-YC4-1-P3-R	TTAATCTTAAATTATTAAATACGTAATT	P3 at *GmNF-YC4-1* promoter used for EMSA
pGmNF-YC4-1-P4-F	TAAAAGAAACAAGATTAAATTACGTAT	P4 at *GmNF-YC4-1* promoter used for EMSA
pGmNF-YC4-1-P4-R	CTTAAGTGAACTAAAGAATCAAATTC	P4 at *GmNF-YC4-1* promoter used for EMSA
pGmNF-YC4-1-P5-F	GGCATTGACATGGATGGATGG	P5 at *GmNF-YC4-1* promoter used for EMSA
pGmNF-YC4-1-P5-R	GGATTACATTGAGTAATGCAC	P5 at *GmNF-YC4-1* promoter used for EMSA
pGmNF-YC4-1-P6-F	CCCTAATATTGTGAACAAAC	P6 at *GmNF-YC4-1* promoter used for EMSA
pGmNF-YC4-1-P6-R	GTGAACTAAAGAATCAAATTCAAAAG	P6 at *GmNF-YC4-1* promoter used for EMSA
pGmNF-YC4-1-P7-F	CATGCTAGAGCAAAGCAAGGT	P7 at *GmNF-YC4-1* promoter used for EMSA
pGmNF-YC4-1-P7-R	CCCCATTGATGGGTCCATTCT	P7 at *GmNF-YC4-1* promoter used for EMSA
GmNF-YC4-1-qPCR-F	CCTCCCAGGCATGGCAGTCC	*GmNF-YC4-1* transcript level
GmNF-YC4-1-qPCR-R	CCATCAAGGCTCCGCTGG	*GmNF-YC4-1* transcript level
GmActin-qPCR-F	GAGCTATGAATTGCCTGATGG	Control gene for transcript level
GmActin-qPCR-R	CGTTTCATGAATTCCAGTAGC	Control gene for transcript level
gRNA 1 GmNF-YC4-1	CATAGTCACATGCCACAACA	gRNA targeting *GmNF-YC4-1* for promoter mutations
gRNA 2 GmNF-YC4-1	CAACAGAGTAACGCTAAACA	gRNA targeting *GmNF-YC4-1* for promoter mutations
gRNA 3 GmNF-YC4-1	CTTTCTTAATTTAAAAACAT	gRNA targeting *GmNF-YC4-1* for promoter mutations
gRNA 4 GmNF-YC4-1	TTTTGTTAACATTAATTTTT	gRNA targeting *GmNF-YC4-1* for promoter mutations
gRNA 5 GmNF-YC4-1	TTATTTGTTGAAGACAAATA	gRNA targeting *GmNF-YC4-1* for promoter mutations
gRNA 6 GmNF-YC4-1	ATATTGTGAACAAACTTTGT	gRNA targeting *GmNF-YC4-1* for promoter mutations
D1-pGmNF-YC4-1-LUC-F1	CCCGGATCCTAATATAATATAAGAATTT GTAAAT	D1 type of deletion for pGmNF-YC4-LUC

D1-pGmNF-YC4-1-LUC-R1	AATACATTTTGTTTAGCGTTACTCATGAGGGTGTTCCATCCATCCATG	D1 type of deletion for pGmNF-YC4-LUC
D1-pGmNF-YC4-1-LUC-F2	CATGGATGGATGGAACACCCTCATGAGTAACGCTAAACAAAATGTATT	D1 type of deletion for pGmNF-YC4-LUC
D1-pGmNF-YC4-1-LUC-R2	CCCAAGCTTTCCCCCAATTTTTTCTGATTTTGA	D1 type of deletion for pGmNF-YC4-LUC
D2-pGmNF-YC4-1-LUC-F1	CCCGGATCCTAATATAATATAAGAATTTGTAAAT	D2 type of deletion for pGmNF-YC4-LUC
D2-pGmNF-YC4-1-LUC-R1	TATTATAAACCTTCCATATTTGTCTTAAATAACCTATGTTTTTAAATTAAGAAAGC	D2 type of deletion for pGmNF-YC4-LUC
D2-pGmNF-YC4-1-LUC-F2	GCTTTCTTAATTTAAAAACATAGGTTATTTAAGACAAATATGGAAGGTTTATAATA	D2 type of deletion for pGmNF-YC4-LUC
D2-pGmNF-YC4-1-LUC-R2	CCCAAGCTTTCCCCCAATTTTTTCTGATTTTGA	D2 type of deletion for pGmNF-YC4-LUC
D3-pGmNF-YC4-1-LUC-F1	CCCGGATCCTAATATAATATAAGAATTTGTAAAT	D3 type of deletion for pOsNF-YC4-LUC
D3-pGmNF-YC4-1-LUC-R1	TAAATATTTAAAAAATCAAATTAAAAAGTTTGTTCACAATATTAGGGAT	D3 type of deletion for pOsNF-YC4-LUC
D3-pGmNF-YC4-1-LUC-F2	ATCCCTAATATTGTGAACAAACTTTTTAATTTGATTTTTTAAATATTTA	D3 type of deletion for pOsNF-YC4-LUC
D3-pGmNF-YC4-1-LUC-R2	CCCAAGCTTTCCCCCAATTTTTTCTGATTTTGA	D3 type of deletion for pOsNF-YC4-LUC
GmCas9-F	GTAGTCAAGGCGGCGAAGTA	Genotype *GmCas9* gene, checking T-DNA insertion
GmCas9-R	CAGGGTTTTCCCAGTCACGA	Genotype *GmCas9* gene, checking T-DNA insertion
GmNF-YC4-1-Del-F	CACGGGCATTGAACAAAAGAGA	Genotype *GmNF-YC4-1* promoter mutations through PCR and sequencing
GmNF-YC4-1-Del-R	AAACCTTCCATATTTGTCTTCAACA	Genotype *GmNF-YC4-1* promoter mutations through PCR and sequencing

New Phytologist

Table S2 Bioinformatic analysis of multiple crop species revealed RAV and WRKY binding motifs were conserved in the promoters of the NF-YC4 orthologs.

Species Name	Common name	AtNF-YC4 homologs	RAV1A motif position (nt)	WRKY motif position (nt)
Chlamydomonas reinhardtii	Algae	Cre12.g556400	-15 to -10; -178 to -183; -270 to -265; -613 to -608; -633 to-628; -714 to -709; -785 to -780	-61 to -56
Micromonas sp RCC299 v3.0	Algae	EuGene.1200010037	-90 to -85; -174 to -169	-11 to -6; -1205 to -1200; -1220 to -1215; -1244 to -1239
Physcomitrium patens	Moss	Pp3c10_2910	-798 to -793; -1442 to -1437	-590 to -585; -1030 to 1025; -1040 to 1035
Arabidopsis thaliana	Arabidopsis	AT5G63470	-771 to -766; -1167 to 1162; -1258 to -1253	-201 to -196; -568 to -563
Solanum tuberosum	Potato	PGSC0003DMG402015259	-374 to -369	-584 to -579; -965 to -960; -1116 to -1111
Brassica napus	Rapeseed	BnaC03g50810D	-1070 to -1065; -1496 to -1491	-951 to -946
Hordeum vulgare	Barley	HORVU6Hr1G032200	N/A	-936 to -931; -1133 to –1128;
Cucumis sativus	Cucumber	Csa_1G001515	-438 to -433; -501 to -496; -955 to -950; -1179 to -1174; -1203 to 1198; -1488 to 1483	-621 to -616
Manihot esculenta	Cassava	MANES_14G050900	-105 to -100; -115 bp to -110; -754 to -749	-823 to -818
Zea mays	Maize	GRMZM2G089812	-297 to -292; -706 to -701	N/A
Gossypium hirsutum	Cotton	Gohir.A12G190000	-1086 to 1081; -1348 to 1343; -1477 to -1472	-416 to -411; -603 to -598; -620 to -615; -1269 to -1264; -1473 to -1468
Solanum lycopersicum	Tomato	Solyc06g072040.1.1	-1180 to -1175; -1346 to -1341	-447 to -442; -730 to -725; -1465 to-1460
Citrus clementina	Clementine	CICLE_v10032646mg	-108 to -103; -117 to -112; -1286 to -1281	-648 to -643; -1247 to 1242
Vitis vinifera	Grape	VIT_17s0000g01130	-542 to -537; -558 to -553; -636 to -631	-561 to -556; -1117 to -1112; -1237 to -1232; -1355 to -1350
Malus x *domestica* 'Golden'	Apple	MD14G0135100	-724 to -719; -751 to -746; -994 to -989	N/A
Musa acuminata		Ma10_g11970	-104 to -99; -120 to -115; -148 to -143; -532 to -527;	-956 to -951; -977 to -972; -1099 to 1094

	Banana		-932 to -927; -1096 to -1091; -1414 to -1409	
Prunus persica	Peach	PRUPE_5G150400	-1140 to -1135; -1203 to -1198; -1270 to -1265; -1339 to -1334; -1397 to -1392; -1439 to -1434	-1043 to -1038; -1143 to -1138; -1148 to -1143
